# High-thermopower polarized electrolytes enabled by methylcellulose for low-grade heat harvesting

**DOI:** 10.1126/sciadv.abl5318

**Published:** 2022-02-18

**Authors:** Yang Han, Jian Zhang, Run Hu, Dongyan Xu

**Affiliations:** 1 Department of Mechanical and Automation Engineering, The Chinese University of Hong Kong, Shatin, New Territories, Hong Kong Special Administrative Region, China.; 2 State Key Laboratory of Coal Combustion, School of Energy and Power Engineering, Huazhong University of Science and Technology, Wuhan 430074, China.

## Abstract

Low-grade heat exists ubiquitously in the environment. Thermogalvanic cells (TGCs) are promising for converting the widespread low-grade heat directly into electricity owing to relatively high thermopowers of redox reactions. This work reports polarized electrolytes with ultrahigh thermopowers of −8.18 mV K^−1^ for n-type and 9.62 mV K^−1^ for p-type. The electrolyte consists of I^−^/I_3_^−^ redox couple, methylcellulose, and KCl. Thermoresponsive methylcellulose leads to polarization switching from n-type to p-type above a transition temperature due to the strong hydrophobic interaction between methylcellulose and I_3_^−^ ions. The giant thermopowers can be attributed to the simultaneously enhanced entropy change and concentration difference of redox couple enabled by the gelation of methylcellulose and KCl-induced complexation. The p-type TGC with the optimized electrolyte achieves a normalized maximum power density of 0.36 mW m^−2^ K^−2^, which is far superior to other reported I^−^/I_3_^−^-based TGCs. This work demonstrates cost-effective, high-thermopower polarized electrolytes for low-grade heat harvesting.

## Teaser

The gelation of methylcellulose and salt-induced complexation enable ultrahigh-thermopower electrolytes for waste heat recovery.

## INTRODUCTION

Low-grade heat is distributed ubiquitously, such as solar heat, body heat, exhaust gas of vehicles, heat dissipated by electrical appliances, and industrial waste heat. Despite its abundance, the utilization of the low-grade heat is very limited due to small temperature differences between heat sources and the ambient environment, fluctuating heat flux, lack of scalable and convenient implementation, etc. (*1*). Liquid thermocells are promising for low-grade heat harvesting due to their merits of the relatively high thermopowers, low cost, and high flexibility (*2*–*5*). Recently, two types of liquid thermocells, namely, thermodiffusion cells (TDCs) (*6*, *7*) and thermogalvanic cells (TGCs) (*8*, *9*), have drawn much attention. On the basis of the Soret effect, a polarized voltage difference is generated in a TDC through thermodiffusion of cations and anions under a temperature gradient (*10*). A TDC works like a capacitor. It can output electric power during the discharging process. Once the charges are fully released, the electric power has to be paused to wait for the next charging process. Although the absolute thermodiffusion thermopower can be as high as 24 mV K^−1^ (*11*), the intermittent working mode of a TDC makes it inconvenient for some application scenarios. In contrast, a TGC is based on electrochemical redox reactions at two symmetric electrodes that are held at different temperatures. The oxidation reaction releases electrons to one electrode, while the reduction reaction absorbs electrons from the other electrode. Such homodromous electron throughput at two electrodes generates continuous electric power, making the TGC more superior and promising for practical applications (*8*, *9*). To date, various redox electrolytes have been reported for TGCs, ranging from organic solvents (*12*, *13*), aqueous electrolytes (*9*, *14*), to ionic liquids (*15*, *16*). A majority of research is focused on aqueous electrolytes due to their advantages of safety and low cost. However, pristine aqueous redox electrolytes exhibit limited thermopowers, for example, I^−^/I_3_^−^ with an absolute thermopower of 0.5 to 0.8 mV K^−1^ (*17*–*20*) and [Fe(CN)_6_]^3−^/[Fe(CN)_6_]^4−^ with an absolute thermopower of 1.4 mV K^−1^ (*14*).

The thermopower of a redox couple is closely related to the entropy change (Δ*S*) and concentration difference (Δ*C*) of redox couple at hot and cold electrodes (note S1). In general, Δ*C* is explicitly close to zero, and thus, most of the research effort is devoted to increasing Δ*S* to boost the thermopower. Δ*S* is mainly enhanced through reorganization of solvent molecules surrounding the redox couple or the structural change of the redox couple (*21*). In this context, Duan *et al.* (*22*) introduced guanidinium and highly soluble urea into the aqueous [Fe(CN)_6_]^3−^/[Fe(CN)_6_]^4−^ electrolyte and boosted the absolute thermopower from 1.4 to 4.2 mV K^−1^. Through the chaotrope-chaotrope ionic bonding interaction, the solvation shells of the redox couple are rearranged, leading to a larger Δ*S* and thereby enhanced thermopower. Kim *et al.* (*13*) explored a more convenient method by simply mixing methanol with water as the solvent of the [Fe(CN)_6_]^3−^/[Fe(CN)_6_]^4−^ electrolyte. The addition of methanol causes the rearrangement of the solvation shells of redox species, which increases the Δ*S* and enhances the absolute thermopower up to 2.9 mV K^−1^ (*13*). Despite achieving high thermopowers, the side effect of adding urea or methanol into the electrolyte in these works (*13*, *22*) is that the ionic conductivity of the electrolyte is largely reduced, which will limit the power performance.

Apart from enlarging Δ*S*, increasing Δ*C* has emerged as another strategy for enhancing the thermopower. An effective approach is to exploit the synergistic confining effects of supramolecules and salts on redox species. The pioneering work of Zhou *et al.* (*18*) used the host-guest interaction between α-cyclodextrin (α-CD) and I^−^/I_3_^−^ redox couple to enhance the thermopower. I_3_^−^ ions are encapsulated by α-CD at low temperatures, leaving a relatively low concentration of free I_3_^−^ ions at the cold electrode. Consequently, the concentration difference of I^−^ and I_3_^−^ ions is high at the cold electrode, thus generating an additional voltage difference according to the Nernst equation. In addition, KCl was added into the α-CD + I^−^/I_3_^−^ electrolyte to assist the formation of the K[α-CD_2_-I_5_] crystal, which further increased Δ*C* and led to an absolute thermopower of 1.97 mV K^−1^. Duan *et al.* (*19*) reported the p-n conversion for the I^−^/I_3_^−^ redox couple by using a thermosensitive nanogel, poly(*N*-isopropylacrylamide) (PNIPAM). PNIPAM has strong hydrophobic interaction with I_3_^−^ ions at high temperatures, which results in a higher Δ*C* at the hot electrode and thus a high absolute thermopower of 1.91 mV K^−1^ (*19*). Recently, Yu *et al.* (*23*) reported that guanidinium cations introduced into the [Fe(CN)_6_]^3−^/[Fe(CN)_6_]^4−^ electrolyte could selectively induce the crystallization of [Fe(CN)_6_]^4−^ ions, which enhances both Δ*C* and Δ*S* and gives rise to a high absolute thermopower of 3.73 mV K^−1^. Han *et al.* (*24*) explored coupling of thermodiffusion and thermogalvanic effects and demonstrated a giant thermopower of 17 mV K^−1^ for ionic gelatin, among which 62.2 and 17.9% came from the thermodiffusion effect of KCl and the thermogalvanic effect of the [Fe(CN)_6_]^3−^/[Fe(CN)_6_]^4−^ redox couple, respectively. Despite great research effort, the thermogalvanic thermopowers of redox couples are still relatively low compared to ionic thermodiffusion thermopowers.

Here, we report thermally induced polarization switching and ultrahigh n-type and p-type thermopowers by introducing methylcellulose (MC) and KCl into the I^−^/I_3_^−^ electrolyte. Different from Han *et al.*’s work (*24*), the thermopower of our ternary electrolyte mainly comes from the thermogalvanic effect of the I^−^/I_3_^−^ redox couple synergistically enhanced by MC and KCl, but the contribution from the thermodiffusion effect of KCl to the thermopower is negligible. In this work, MC is chosen as the complexation agent due to its abundance, low cost, biocompatibility (*25*), and temperature-dependent switching between hydrophilic and hydrophobic states (*26*, *27*). The hydrophobic interaction between MC and I_3_^−^ ions leads to a substantially reduced I_3_^−^ concentration at the hot electrode. When the hot electrode temperature is above the gelation temperature of MC, polarization switching from n-type to p-type is observed for the I^−^/I_3_^−^ redox couple due to the reversed redox reactions at two electrodes. The I^−^/I_3_^−^ + MC binary electrolytes show a maximum n-type thermopower of −1.32 mV K^−1^ and a maximum p-type thermopower of 1.48 mV K^−1^, respectively, compared to −0.71 mV K^−1^ for the pristine I^−^/I_3_^−^ electrolyte. Unexpectedly, the addition of KCl substantially boosts both n-type and p-type thermopowers of the I^−^/I_3_^−^ + 2 weight % (wt %) MC binary electrolyte. The optimized ternary electrolyte, I^−^/I_3_^−^ + 2 wt % MC + 0.3 M KCl, yields thermopowers as high as −8.18 mV K^−1^ for n-type and 9.62 mV K^−1^ for p-type, both of which are one order of magnitude higher than the thermopower of the pristine I^−^/I_3_^−^ electrolyte and the highest thermogalvanic thermopowers reported so far. Moreover, the transition temperature is lowered from 56° to 32°C depending on the KCl content, indicating that KCl is effective for tuning the electrolyte polarization. Materials characterization results indicate that both Δ*C* and Δ*S* are enhanced, arising from the gelation of MC and KCl-induced complexation. With the optimized electrolyte, we further demonstrated that single n-type and p-type TGCs could generate a maximum power of 27.78 and 80.47 μW, respectively, under a temperature difference of 15°C. This work bridges biopolymers and TGCs and opens new opportunities for a wide class of low-cost biopolymers to be applied in liquid thermocells for low-grade heat harvesting.

## RESULTS

### I^−^/I_3_^−^ + *x* MC binary electrolytes

**Fig. 1.**
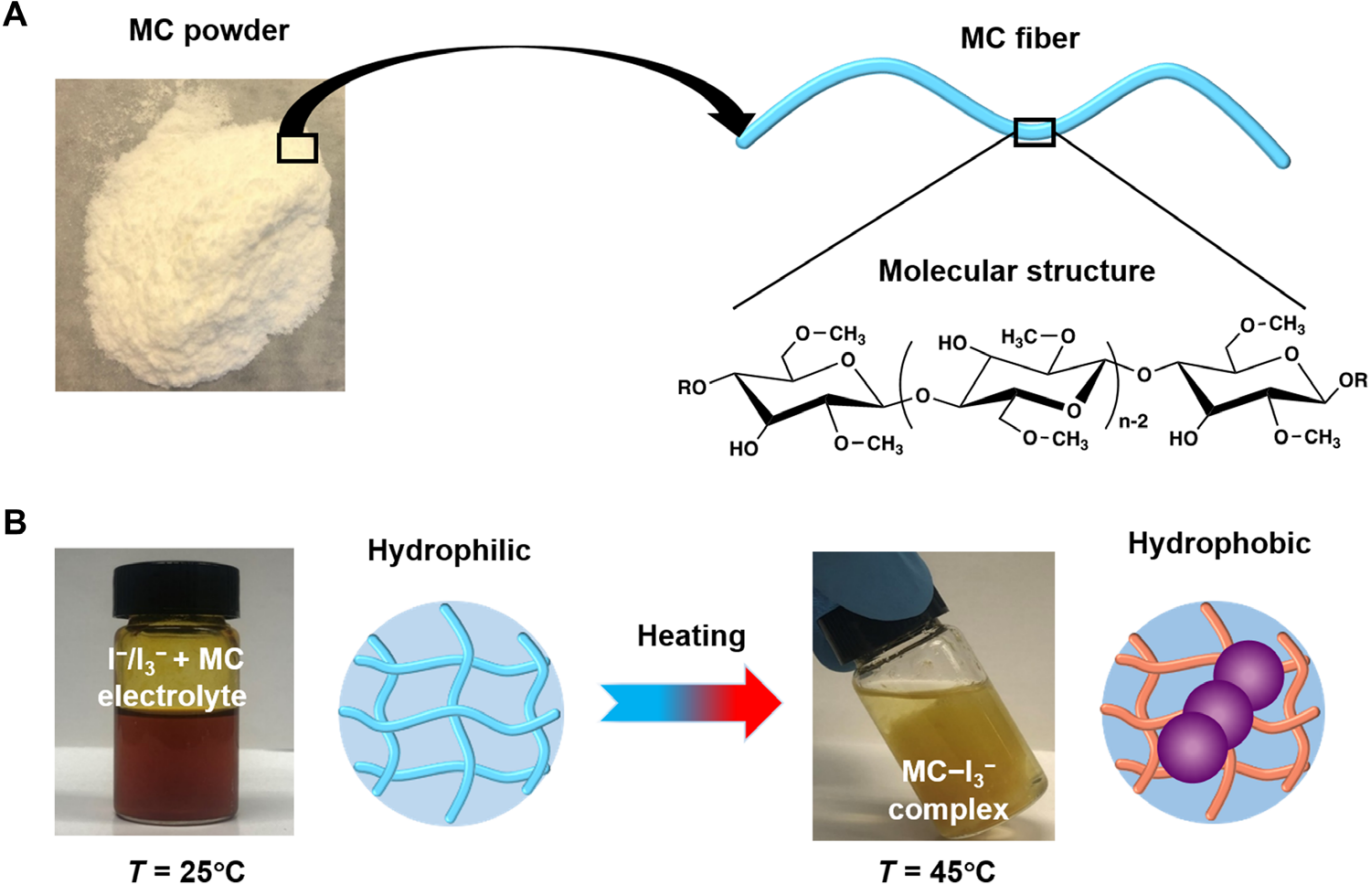
Interactions between MC and the I^−^/I_3_^−^ electrolyte. (**A**) Photograph of MC powder, schematic of an MC fiber, and the molecular structure of MC. (**B**) Photographs of the I^−^/I_3_^−^ + MC electrolyte at different temperatures and the schematics showing the change of MC from the hydrophilic to hydrophobic state upon heating. I_3_^−^ ions interact with MC through hydrophobic interaction at high temperatures. Photo credit: (A and B) Yang Han, The Chinese University of Hong Kong.

MC is a cellulose derivative with partial hydroxyl groups (─OH) of anhydro-d-glucose units substituted by methoxy groups (─OCH_3_) (Fig. 1A). MC is water-soluble at low temperatures. Upon heating, the hydrophobic methyl groups (─CH_3_) of MC are exposed, inducing the formation of aggregates (*26*, *27*). I_3_^−^ ions are relatively more hydrophobic than I^−^ ions, which will preferentially interact with methyl groups of MC through the hydrophobic interaction (*19*, *28*). Figure 1B indicates that the MC-I_3_^−^ complex is formed upon heating as the solution color changes from dark brown (I_3_^−^) to colorless (I^−^) (*19*). The interaction between MC and I_3_^−^ ions is also confirmed by the Fourier transform infrared (FTIR) spectroscopy. Compared to the pure MC, the C─H stretching bands at 2920 and 940 cm^−1^ are greatly weakened for the I^−^/I_3_^−^ + MC binary electrolyte, indicating the hydrophobic interaction between methyl groups of MC and I_3_^−^ ions (fig. S1), which is consistent with the previous work (*29*).

**Fig. 2.**
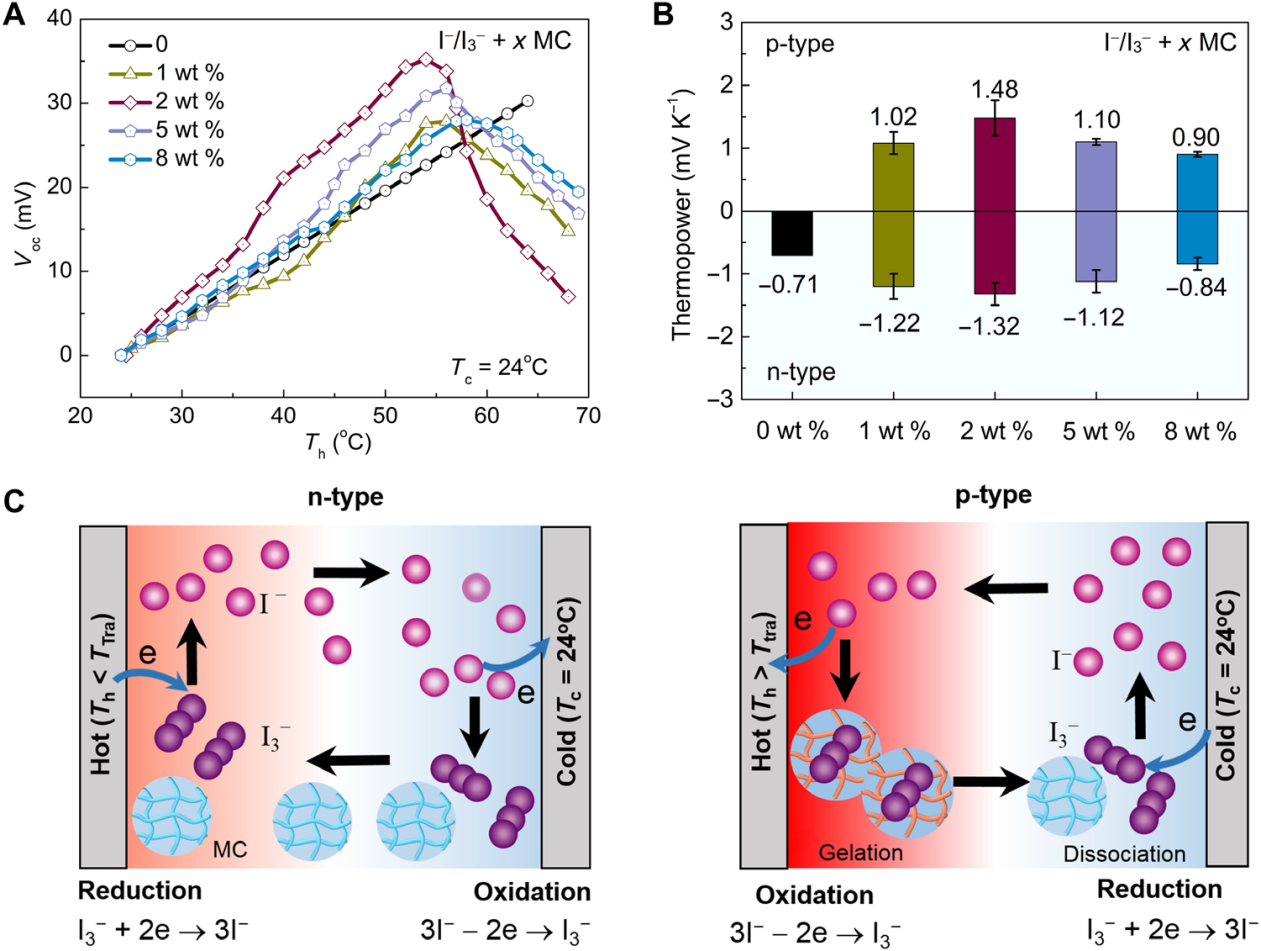
Thermopower enhancement and polarization switching from n-type to p-type for the I^−^/I_3_^−^ redox couple induced by MC. (**A**) Recorded open-circuit voltages with the increase of the hot electrode temperature for the I^−^/I_3_^−^ + *x* MC electrolytes (*x* = 0, 1, 2, 5, and 8 wt %). The cold electrode temperature is held at 24°C. (**B**) Extracted mean thermopowers of the I^−^/I_3_^−^ + *x* MC electrolytes. (**C**) Schematics of the polarization switching from n-type (*T*_h_ < *T*_Tra_) to p-type (*T*_h_ > *T*_Tra_) induced by the gelation of MC.

The thermopowers of the I^−^/I_3_^−^ + *x* MC electrolytes were characterized with a homemade setup (fig. S2). Before testing, 15 ml of electrolyte was filled in the cavity. A quasi–steady-state differential method is adopted to determine the thermopower, which is similar to the Seebeck coefficient measurement of thermoelectric materials (*30*). During the thermopower measurement, the cold electrode was held at 24°C; the open-circuit voltage (*V*_oc_) was recorded when the hot electrode was heated up slowly. Figure 2A shows the recorded open-circuit voltages with the increase of the hot electrode temperature (*T*_h_) for the I^−^/I_3_^−^ electrolytes with various MC contents (*x* = 0, 1, 2, 5, and 8 wt %). For the pristine I^−^/I_3_^−^ electrolyte (*x* = 0), the open-circuit voltage increases linearly with the hot electrode temperature. The thermopower can be determined from the slope of the *V*_oc_-*T*_h_ curve, which is −0.71 mV K^−1^ for the pristine I^−^/I_3_^−^ electrolyte. It is worth noting that the definition of temperature coefficient in electrochemistry has opposite sign convention from Seebeck coefficient in thermoelectrics (*4*). Recently, Han *et al.* (*24*) discussed the sign conventions of thermogalvanic and thermodiffusion thermopowers, and they followed the sign convention of Seebeck coefficient for the definition of thermopower. Here, the same sign convention is adopted and the thermopower is defined as *S*_TGC_ = −Δ*V*/Δ*T*. Therefore, the pristine I^−^/I_3_^−^ electrolyte has a negative thermopower and is defined as n-type. As seen in table S1, the thermopower of the aqueous I^−^/I_3_^−^ electrolyte slightly increases with the decrease of the I^−^/I_3_^−^ concentration. In this work, the I^−^/I_3_^−^ concentration is fixed at 5 mM, which is close to the optimal concentration with the highest thermopower. The absolute thermopower we determined for the I^−^/I_3_^−^ redox couple is close to those reported in the literature (*18*, *19*). For the binary electrolytes, the open-circuit voltage first increases with temperature, reaches a maximum at ~56°C, and then drops at higher temperatures (Fig. 2A), which implies that MC enables thermally induced polarization switching from n-type to p-type for I^−^/I_3_^−^ redox couple above a transition temperature (*T*_Tra_). The gelation temperature of MC was determined by the micro–differential scanning calorimetry (DSC) measurement (fig. S3). The extracted gelation temperature of MC (57°C) agrees well with the n-p transition temperature (~56°C) of the binary electrolytes, indicating that polarization switching is induced by the gelation of MC. The mean thermopowers in the n-type and p-type temperature regions are depicted in Fig. 2B, and the variance of the thermopower is shown as the error bar. As shown in Fig. 2B, the binary electrolytes demonstrate higher absolute thermopowers than the pristine electrolyte, and the I^−^/I_3_^−^ + 2 wt % MC electrolyte achieves the highest values, with an n-type thermopower of −1.32 mV K^−1^ and a p-type thermopower of 1.48 mV K^−1^.

The polarization switching can be attributed to the reversed reactions at two electrodes induced by the hydrophobic interaction between MC and I_3_^−^ ions at the hot electrode (Fig. 2C and fig. S4, A and B). As depicted in Fig. 2C, MC behaves distinctly at two electrodes. Because of its thermoresponsive nature, MC gradually becomes hydrophobic at the hot electrode but remains hydrophilic at the cold electrode. As the hot electrode is heated up, MC and I_3_^−^ ions form complexes through the hydrophobic interaction, resulting in a lower concentration of free I_3_^−^ ions. To investigate the concentration changes of I^−^ and I_3_^−^ ions, the ultraviolet-visible (UV-Vis) spectroscopy characterization was conducted on both the pristine I^−^/I_3_^−^ and I^−^/I_3_^−^ + 2 wt % MC electrolytes, and the UV-Vis spectra are shown in fig. S5. At the hot electrode, the I_3_^−^ intensity sharply decreases, while the I^−^ signal remains unchanged for the I^−^/I_3_^−^ + 2 wt % MC electrolyte, which confirms that MC lowers the I_3_^−^ concentration. When the hot electrode temperature is above the transition temperature (~56°C), the concentration of I_3_^−^ ions is much lower than that of I^−^ ions and the reversed reactions will occur at two electrodes. For *T*_h_ < *T*_Tra_ (n-type) and *T*_h_ > *T*_Tra_ (p-type), the reactions at hot and cold electrodes are listed as follows

**Table:** 

n-type	Hot electrode:	I_3_^−^ + 2e → 3I^−^
	Cold electrode:	3I^−^ − 2e → I_3_^−^
p-type	Hot electrode:	3I^−^ − 2e → I_3_^−^
	Cold electrode:	I_3_^−^ + 2e → 3I^−^

As mentioned above, MC interacts with I_3_^−^ ions at elevated temperatures and thus leads to a larger concentration difference of I_3_^−^ ions between two electrodes. On the other hand, the electrolyte is in the form of solution at low temperatures. Upon heating, MC gradually turns into gelation, which forces the reaction to proceed in a quasi-solid medium. As a result, Δ*S* is also increased because of the change of the solvation environment. Therefore, the enhanced thermopowers of the binary electrolytes can be attributed to the simultaneously increased Δ*C* and Δ*S* induced by the phase transition of MC (note S1). Although MC can enhance the thermopower of the I^−^/I_3_^−^ electrolyte, 5 wt % MC tends to solidify the electrolyte (fig. S6A). Besides, the cyclic voltammetry (CV) measurements show that MC hinders the electrochemical activities of the electrolyte (fig. S6, B and C). The peak separation (*E*_pp_) of a CV curve reflects the electron transfer kinetics. Compared to the pristine I^−^/I_3_^−^ electrolyte with *E*_pp_ = 220 mV, the I^−^/I_3_^−^ + 2 wt % MC electrolyte shows a larger *E*_pp_ of 320 mV, indicating the inferior redox reaction. In addition, MC also leads to a lower current density (fig. S6C). As a result, the thermopower of the I^−^/I_3_^−^ + *x* MC electrolytes is optimized at *x* = 2 wt %. The MC content is fixed at 2 wt % for the following study.

### I^−^/I_3_^−^ + 2 wt % MC + *y* KCl ternary electrolytes

**Fig. 3.**
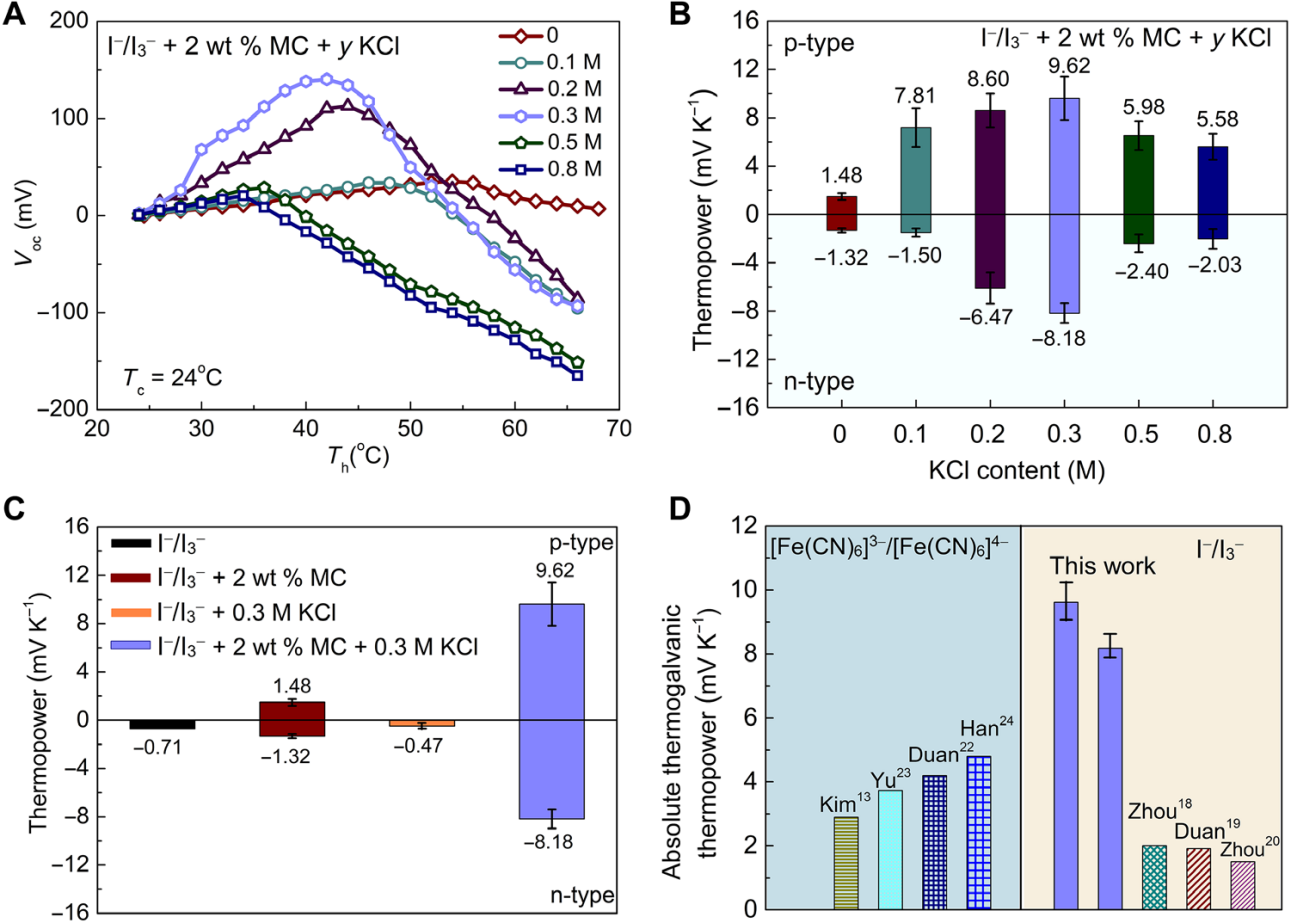
Giant thermopowers of the I^−^/I_3_^−^+ 2 wt % MC + *y* KCl ternary electrolytes. (**A**) Recorded open-circuit voltages with the increase of the hot electrode temperature for the ternary electrolytes with various KCl contents (*y* = 0, 0.1, 0.2, 0.3, 0.5, and 0.8 M). The cold electrode temperature is held at 24°C. (**B**) Extracted mean thermopowers of the I^−^/I_3_^−^ + 2 wt % MC + *y* KCl electrolytes. (**C**) Comparison of the thermopowers of the pristine I^−^/I_3_^−^, I^−^/I_3_^−^ + 2 wt % MC, I^−^/I_3_^−^ + 0.3 M KCl, and I^−^/I_3_^−^ + 2 wt % MC + 0.3 M KCl electrolytes. (**D**) Comparison of the absolute thermogalvanic thermopowers of the optimized ternary electrolyte with those reported in the literature for I^−^/I_3_^−^ and [Fe(CN)_6_]^3−^/[Fe(CN)_6_]^4−^ redox couples.

Similar to Zhou *et al.*’s work (*18*), KCl was added into the I^−^/I_3_^−^ + 2 wt % MC electrolyte to further enhance the thermopower. The open-circuit voltages of the ternary electrolytes with various KCl contents (*y* = 0, 0.1, 0.2, 0.3, 0.5, and 0.8 M) are shown in Fig. 3A as a function of the hot electrode temperature, while the cold electrode is held at 24°C. The extracted mean thermopowers are summarized in Fig. 3B. Unexpectedly, KCl substantially boosts both n-type and p-type thermopowers of the ternary electrolytes. Especially, with 0.3 M KCl, we achieved remarkably high thermopowers of −8.18 mV K^−1^ for n-type and 9.62 mV K^−1^ for p-type (Fig. 3B). To verify the results, the steady-state thermopower measurement was also conducted on the I^−^/I_3_^−^ + 2 wt % MC + 0.3 M KCl electrolyte. Both n-type and p-type thermopowers obtained by the steady-state method are consistent with the mean thermopowers extracted by the quasi–steady-state differential method (fig. S7). When the KCl content is beyond 0.3 M, the ternary electrolyte becomes unstable and precipitates at room temperature (fig. S8). Since the thermodiffusion effect of mobile ions (K^+^ and Cl^−^) in the ternary electrolyte will also contribute to the thermopower (fig. S3C), we further characterized the thermodiffusion thermopowers of the pure KCl and the 2 wt % MC + *y* KCl electrolytes. The absolute thermodiffusion thermopowers are lower than 0.4 mV K^−1^ (fig. S9B), indicating the marginal contribution to the overall thermopower. The thermopower of the I^−^/I_3_^−^ + 0.3 M KCl electrolyte was also measured for comparison. As shown in Fig. 3C, the I^−^/I_3_^−^ + 0.3 M KCl electrolyte has a thermopower of −0.47 mV K^−1^, which is slightly lower than the thermopower of the pristine I^−^/I_3_^−^ electrolyte presumably due to the competition between the n-type thermogalvanic effect of I^−^/I_3_^−^ redox couple and the p-type thermodiffusion effect of KCl. It can be concluded that the giant thermopowers of our ternary electrolyte mainly come from the thermogalvanic effect of I^−^/I_3_^−^ redox couple enhanced by MC and KCl. As shown in Fig. 3D, both n-type and p-type thermopowers of the optimized ternary electrolyte outperform those reported in the literature for I^−^/I_3_^−^ and [Fe(CN)_6_]^3−^/[Fe(CN)_6_]^4−^ redox couples (*13*, *18*–*20*, *22*–*24*).

Besides, KCl effectively reduces the transition temperature for the electrolyte to switch from n-type to p-type as seen in Fig. 3A. The n-p transition temperature is 56°C for the I^−^/I_3_^−^ + 2 wt % MC binary electrolyte, while it decreases monotonically from 50° to 32°C when the KCl content increases from 0.1 to 0.8 M. Table 1 compares the gelation and n-p transition temperatures for the ternary electrolytes, which agree reasonably well with each other. The reduction in the transition temperature can be attributed to the salting out effect of kosmotropic Cl^−^ anions (*31*). In general, Cl^−^ ions are considered as structure making ions (*32*) and have strong interaction with water molecules. As a result, minor heating will cause the destruction of the MC-water structure and the exposure of hydrophobic groups of MC, thus leading to a lower transition temperature for the ternary electrolyte (*31*, *33*).

**Table 1.** Comparison of the gelation and n-p transition temperatures for the I^−^/I_3_^−^+ 2 wt % MC + *y* KCl electrolytes (*y* = 0.1, 0.2, 0.3, 0.5, and 0.8 M).

	**Gelation** **temperature (°C)**	**n-p transition** **temperature (°C)**
I^−^/I_3_^−^ + 2 wt % MC + 0.1 M KCl	52	50
I^−^/I_3_^−^ + 2 wt % MC + 0.2 M KCl	45	44
I^−^/I_3_^−^ + 2 wt % MC + 0.3 M KCl	44	43
I^−^/I_3_^−^ + 2 wt % MC + 0.5 M KCl	39	36
I^−^/I_3_^−^ + 2 wt % MC + 0.8 M KCl	37	32

**Fig. 4.**
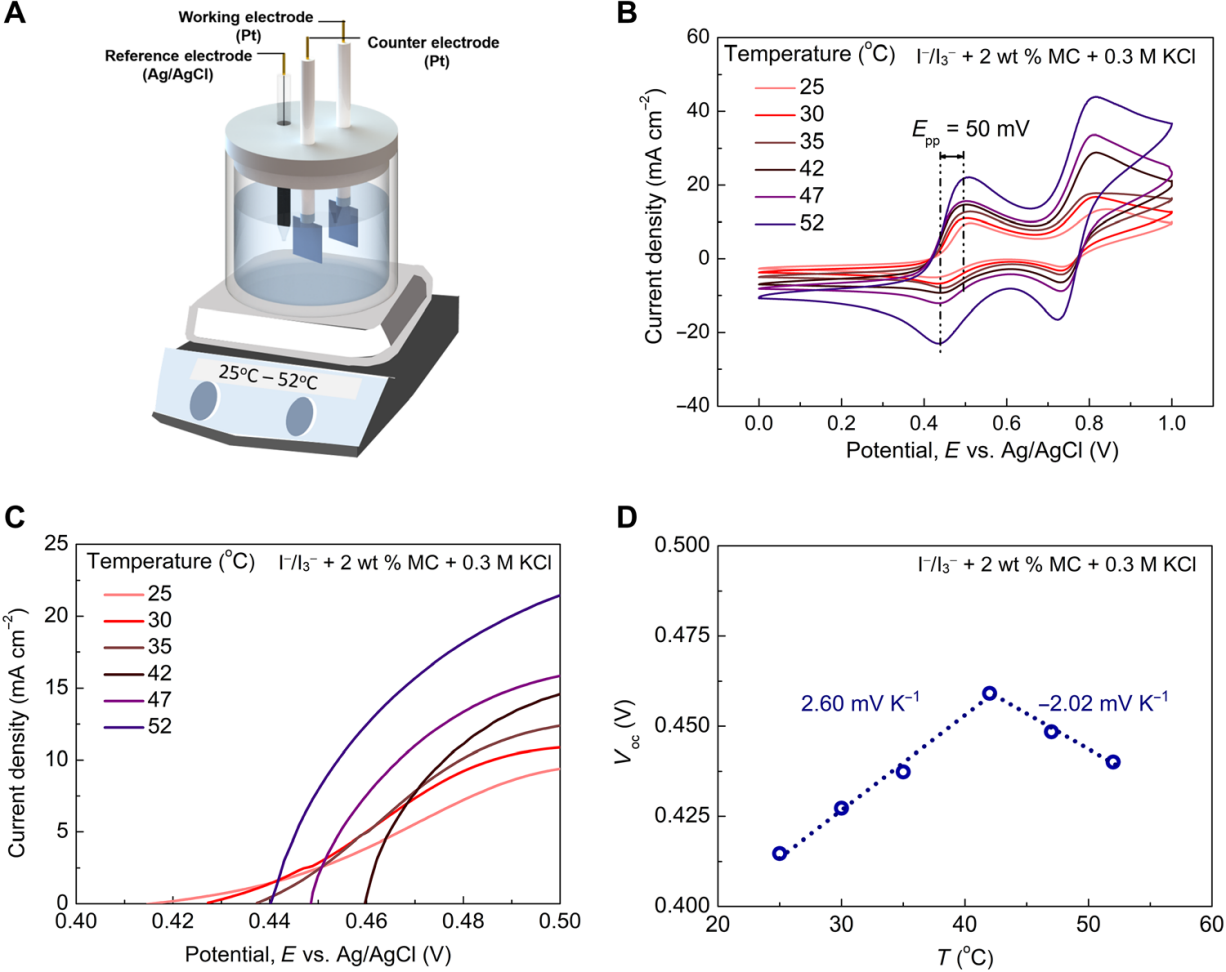
Temperature coefficient of the redox reaction for the I^−^/I_3_^−^+ 2 wt % MC + 0.3 M KCl electrolyte. (**A**) Schematic of the three-electrode configuration for isothermal CV measurements. (**B**) CV curves in the temperature range from 25° to 52°C. (**C**) Open-circuit voltages under different temperatures. (**D**) Temperature dependence of the open-circuit voltage. The slope of the linear fitting curve gives the temperature coefficient of the redox reaction.

To understand the mechanism of giant thermopowers, we first characterized the temperature coefficient of the redox reaction for the I^−^/I_3_^−^ + 2 wt % MC + 0.3 M KCl electrolyte. Temperature-dependent CV measurements were conducted under an isothermal three-electrode configuration as shown in Fig. 4A. Figure 4 (B and C) shows the CV curves and open-circuit voltages (*x*-axis intercept), in the temperature range from 25° to 52°C. Figure 4D plots the temperature dependence of the open-circuit voltage, which shows a first increasing and then decreasing trend, indicating the transition from n-type to p-type. The temperature coefficients of the ternary electrolyte can be determined from the slope of the linear fitting curve, which are 2.60 and −2.02 mV K^−1^ for the n-type and p-type temperature regions, respectively. As mentioned above, the temperature coefficient and thermopower have opposite signs due to different sign conventions. It is worth noting that the temperature coefficient of the Ag/AgCl reference electrode is negligible (*34*). The absolute temperature coefficients (2.60 and 2.02 mV K^−1^) are much lower than the absolute thermopowers (8.18 and 9.62 mV K^−1^) for both n-type and p-type temperature regions. As shown in Eq. *7* (note S1), in addition to the temperature coefficient, Δ*S* and Δ*C* of redox species induced by the phase transition of MC and the addition of KCl will also contribute to the thermopower of the ternary electrolyte. Furthermore, the CV curves of the I^−^/I_3_^−^ + 2 wt % MC + 0.3 M KCl electrolyte (Fig. 4B) show an *E*_pp_ of 50 mV, which is much smaller than the counterpart of the I^−^/I_3_^−^ + 2 wt % MC electrolyte (*E*_pp_ = 320 mV), indicating the faster electron transfer kinetics and the better reversibility.

**Fig. 5.**
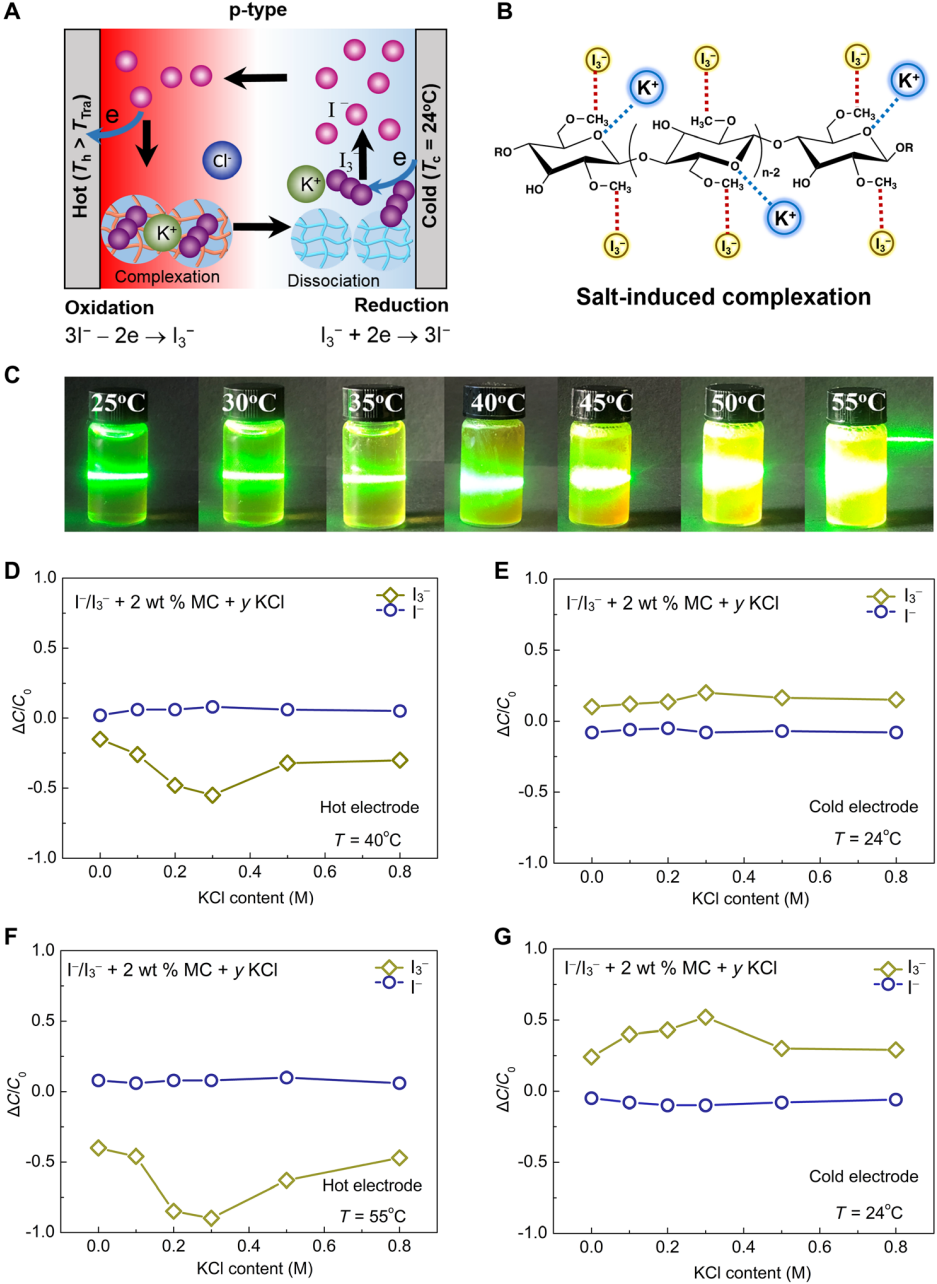
Salt-induced complexation enabling high thermopowers of the ternary electrolytes. Schematics of (**A**) a p-type TGC with the ternary electrolyte and (**B**) salt-induced complexation. K^+^ and I_3_^−^ ions interact with MC through O sites and methyl groups, respectively. (**C**) Turbidity of the I^−^/I_3_^−^ + 2 wt % MC + 0.3 M KCl electrolyte under different temperatures. Relative concentration changes (Δ*C*/*C*_0_) of I^−^ and I_3_^−^ ions at two electrodes for TGCs with a hot electrode temperature of (**D** and **E**) 40°C and (**F** and **G**) 55°C. Photo credit: (C) Yang Han, The Chinese University of Hong Kong.

To understand the effect of KCl on thermopowers of the ternary electrolytes, we further investigated the interaction between KCl and the I^−^/I_3_^−^ + 2 wt % MC electrolyte. It is well known that MC has lone pair electrons at oxygen (O) sites on the glucose ring and methoxy groups, which can serve as complexation sites for cations (*35*). The complexation between MC and cations such as K^+^ ions will result in the decrease of crystallinity for MC (*36*). To confirm the interaction between K^+^ ions and MC in the electrolyte, x-ray diffraction (XRD) characterization was conducted on both pure MC and complexes of MC and KCl. For the dried 2 wt % MC + 0.3 M KCl powders, two relatively broad peaks in XRD pattern decrease markedly (fig. S10A), indicating that the semi-crystalline MC becomes more amorphous after complexation. FTIR characterization further shows that the characteristic peak for the stretching mode of the ─OCH_3_ bond at 1048 cm^−1^ broadens and weakens for the I^−^/I_3_^−^ + 2 wt % MC + *y* KCl electrolytes (fig. S10B), which suggests that K^+^ ions interact with MC at O sites. It has been reported that KCl leads to the aggregation of supramolecular α-CD in the presence of I_3_^−^ ions, forming the K[α-CD_2_-I_5_] crystal (*18*). Similarly, KCl induces complexation with MC in the ternary electrolyte. MC forms the complex with K^+^ and I_3_^−^ ions along its fibril backbone, which favors the reversible complexation and dissociation processes. Figure 5A shows the schematic of a p-type TGC with the ternary electrolyte. Upon heating, K^+^ and I_3_^−^ ions interact with MC through O sites and methyl groups, respectively, forming the K^+^-MC-I_3_^−^ complex (Fig. 5B). Figure 5C shows the turbidity of the I^−^/I_3_^−^ + 2 wt % MC + 0.3 M KCl electrolyte under different temperatures. The complex precipitates above 40°C and dissociates after cooling, indicating that it turns into solution at the cold electrode. The precipitation-dissociation process of the K^+^-MC-I_3_^−^ complex will lead to the large entropy change of the I^−^/I_3_^−^ redox couple (*23*). In addition to the entropy change, we conducted UV-Vis spectroscopy to characterize the relative concentration changes of redox species at hot and cold electrodes for both n-type and p-type TGCs. The absorbance profiles of I^−^ and I_3_^−^ ions are plotted in fig. S11 for the ternary electrolytes with various KCl contents. Figure S11 shows that the concentration of I^−^ ions remains almost unchanged, while the concentration of I_3_^−^ ions varies substantially depending on the KCl content. The relative concentration changes (Δ*C*/*C*_0_) of I^−^ and I_3_^−^ ions derived from the absorbance profiles are plotted in Fig. 5 (D to G). As seen in Fig. 5 (D and F), the concentration of I_3_^−^ ions at the hot electrode decreases because of the complexation with MC, while KCl further reduces the I_3_^−^ concentration with the largest Δ*C*/*C*_0_ observed at 0.3 M. For the n-type TGC with 0.3 M KCl (Fig. 5D), the Δ*C*/*C*_0_ of I_3_^−^ ions is −0.6 at the hot electrode, while the I_3_^−^ concentration at the cold electrode shows an opposite increasing trend with Δ*C*/*C*_0_ = 0.3 (Fig. 5E). The unbalanced concentration changes of I_3_^−^ ions may result from the rapid precipitation and dissociation of the K^+^-MC-I_3_^−^ complex at two electrodes. On the other hand, even larger I_3_^−^ concentration changes are observed for the p-type TGC with 0.3 M KCl, with the largest Δ*C*/*C*_0_ of −0.9 and 0.6 at hot and cold electrodes (Fig. 5, F and G), respectively. Because of the substantially enhanced Δ*S* and Δ*C* caused by KCl-induced complexation for both n-type and p-type TGCs, our ternary electrolyte achieves record-high thermopowers of −8.18 mV K^−1^ for n-type and 9.62 mV K^−1^ for p-type at the KCl content of 0.3 M.

### Power generation performance of single n-type and p-type TGCs

**Fig. 6.**
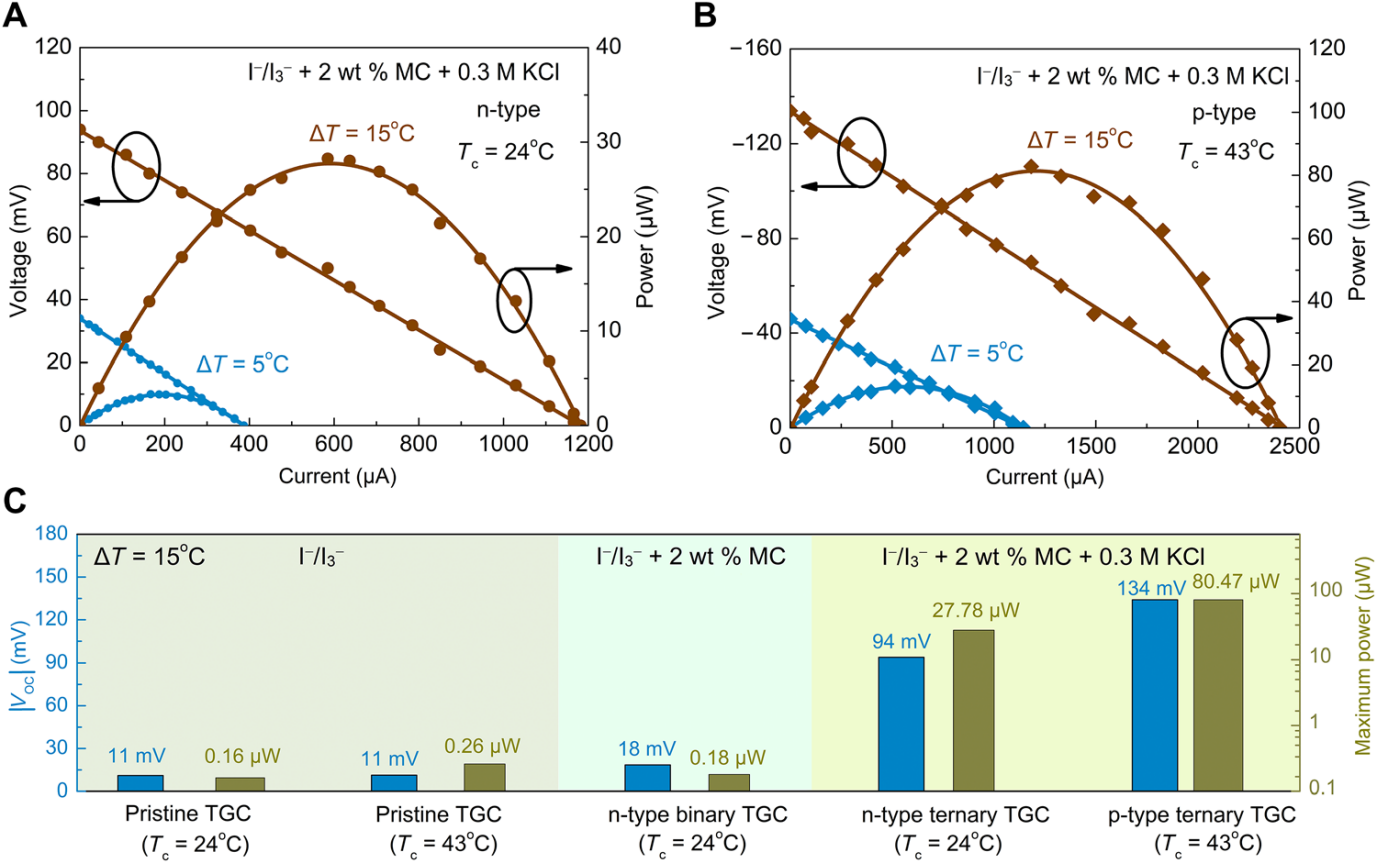
Power generation performance of single n-type and p-type TGCs. Voltage-current curves and output powers of (**A**) n-type and (**B**) p-type TGCs with the optimized ternary electrolyte under Δ*T* = 5° and 15°C. The cold electrode temperature is held at 24° and 43°C for the n-type and p-type ternary TGCs, respectively. (**C**) Comparison of the open-circuit voltages and maximum output powers for the TGCs with the pristine, binary, and ternary electrolytes.

With the optimized I^−^/I_3_^−^ + 2 wt % MC + 0.3 M KCl electrolyte, we further characterized the output powers of single n-type and p-type TGCs under temperature differences (Δ*T*) of 5° and 15°C, respectively. The cold electrode temperature (*T*_c_) of the n-type TGC is fixed at 24°C. The ternary electrolyte with 0.3 M KCl switches from n-type to p-type when the hot electrode temperature is above 43°C (Table 1). To fully exploit the high p-type thermopower of the ternary electrolyte, the cold electrode temperature is kept at 43°C for the p-type TGC. Figure 6 (A and B) plots the voltage-current curves and output powers for n-type and p-type TGCs, respectively, under two temperature differences. For comparison, the output powers of the TGCs filled with the pristine I^−^/I_3_^−^ and I^−^/I_3_^−^ + 2 wt % MC binary electrolytes were also characterized under Δ*T* = 15°C, as shown in fig. S12. Figure 6C shows the comparison of the open-circuit voltages and maximum output powers for the TGCs with the pristine, binary, and ternary electrolytes. As seen in Fig. 6C, under Δ*T* = 15°C, our n-type and p-type TGCs with the optimized ternary electrolyte achieve maximum powers of 27.78 and 80.47 μW, respectively, both of which are much larger than the counterparts of the TGCs with the pristine (0.16 μW for *T*_c_ = 24°C and 0.26 μW for *T*_c_ = 43°C) and binary electrolytes (0.18 μW for *T*_c_ = 24°C). The durability of the TGCs was evaluated by monitoring the open-circuit voltage under a constant temperature difference for a relatively long period (fig. S13). Both n-type and p-type ternary TGCs can maintain a stable output voltage under Δ*T* = 15°C, confirming the durability of our TGCs.

The enhanced maximum powers of both n-type and p-type ternary TGCs can be attributed to the simultaneously enhanced thermopower and ionic conductivity of the ternary electrolyte. Under Δ*T* = 15°C, the n-type and p-type TGCs give rise to open-circuit voltages of 94 and −134 mV, respectively. The absolute open-circuit voltages are much higher than that of the pristine TGC (11 mV) due to the substantially enhanced thermopowers of the ternary electrolyte. In addition, the short-circuit currents are 1.18 and 2.40 mA, respectively, for the n-type and p-type TGCs under Δ*T* = 15°C, while it is only 0.06 mA for the pristine TGC. The large current output of the n-type and p-type TGCs can be explained by the high ionic conductivity of the optimized ternary electrolyte (fig. S14) and thus the low internal resistance (fig. S15). At room temperature, the pristine I^−^/I_3_^−^ electrolyte shows a poor ionic conductivity of ~1.2 mS cm^−1^. In a sharp contrast, the ionic conductivity of the I^−^/I_3_^−^ + 2 wt % MC + 0.3 M KCl electrolyte reaches 36 mS cm^−1^ at 300 K, which is 30 times the value of the pristine electrolyte. As a result, the internal resistance of n-type and p-type TGCs is much lower than the counterpart of the pristine TGC. As seen in fig. S15, the pristine TGC exhibits the internal resistance of 122 ohms under Δ*T* = 15°C, while the corresponding value is only 19 ohms for the n-type TGC and 17 ohms for the p-type one. Figure S14 also shows that the ionic conductivity of the optimized ternary electrolyte slightly increases with temperature, which leads to the lower internal resistance of the p-type TGC.

## DISCUSSION

**Fig. 7.**
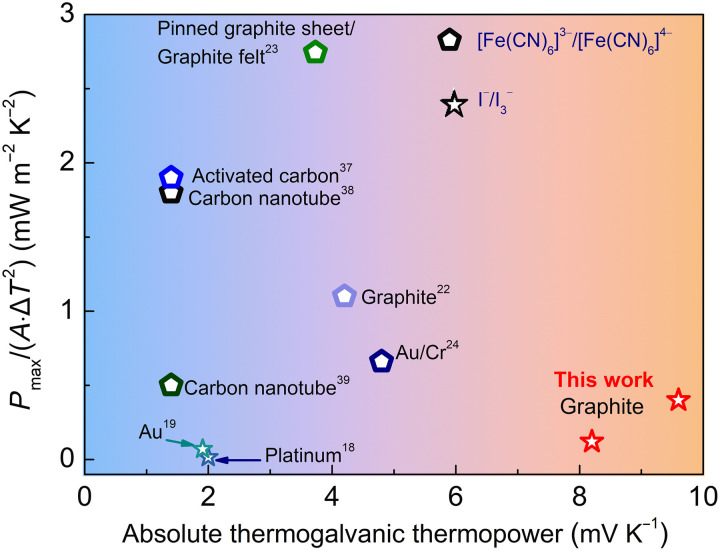
Comparison of the absolute thermogalvanic thermopowers and normalized maximum power densities of n-type and p-type ternary TGCs in this work with those reported for [Fe(CN)_6_]^3−^/[Fe(CN)_6_]^4−^-based and I^−^/I_3_^−^-based thermocells in the literature.

Figure 7 and Table 2 compare the absolute thermogalvanic thermopowers and normalized maximum power densities, *P*_max_/(*A*·Δ*T*^2^), of our n-type and p-type TGCs with those reported for [Fe(CN)_6_]^3−^/[Fe(CN)_6_]^4−^-based and I^−^/I_3_^−^-based thermocells in the literature (*18*, *19*, *22*–*24*, *37*–*39*). The electrode materials used in those works are also listed for comparison. As shown in Figs. 3D and 7, our electrolyte achieves the highest absolute thermogalvanic thermopowers among the reported works. The normalized maximum power densities of our n-type and p-type TGCs reach 0.12 and 0.36 mW m^−2^ K^−2^, respectively, which outperform other I^−^/I_3_^−^-based TGCs in the literature (*18*, *19*). On the other hand, [Fe(CN)_6_]^3−^/[Fe(CN)_6_]^4−^-based thermocells demonstrate much higher normalized maximum power density, up to 2.74 mW m^−2^ K^−2^ (*23*), which can be attributed to the high ionic conductivity of [Fe(CN)_6_]^3−^/[Fe(CN)_6_]^4−^-based electrolytes and the improvement of electrode materials. It has been shown that electrode materials with high electrical conductivity (*23*), high surface area (*23*, *38*, *39*), and catalytic capabilities (*24*) can effectively improve the current density and thus output power of liquid thermocells. Graphite is used as electrode materials in this work. The normalized maximum power density of our TGCs might be further enhanced through the electrode modification.

**Table 2.** Comparison of |*S*_TGC_| and *P*_max_/(*A*·Δ*T*^2^) in this work with those reported for [Fe(CN)_6_]^3−^/[Fe(CN)_6_]^4−^-based and I^−^/I_3_^−^-based thermocells in the literature.

	**Electrolyte**	**Electrode**	**|*S*_TGC_|** **(mV K^−1^)**	***P*_max_/** **(*A*·Δ*T*^2^)** **(mW** **m^−2^ K^−2^)**
Duan *et al.* (*22*)	[Fe(CN)_6_]^3−^/ [Fe(CN)_6_]^4−^	Graphite	4.2	1.1
Yu *et al.* (*23*)	[Fe(CN)_6_]^3−^/ [Fe(CN)_6_]^4−^	Graphite felt/pinned graphite sheet	3.73	2.74
Han *et al.* (*24*)	[Fe(CN)_6_]^3−^/[Fe(CN)_6_]^4−^	Au/Cr	4.8	0.66
Zhang *et al.* (*37*)	[Fe(CN)_6_]^3−^/ [Fe(CN)_6_]^4−^	Activated carbon	1.4	1.9
Im *et al.* (*38*)	[Fe(CN)_6_]^3−^/ [Fe(CN)_6_]^4−^	Carbon nanotube	1.4	1.8
Hu *et al.* (*39*)	[Fe(CN)_6_]^3−^/ [Fe(CN)_6_]^4−^	Carbon nanotube	1.4	0.5
Zhou *et al.* (*18*)	I^−^/I_3_^−^	Platinum	2	0.015
Duan *et al.* (*19*)	I^−^/I_3_^−^	Au	1.91	0.07
This work	I^−^/I_3_^−^	Graphite	8.18 (n-type)	0.12 (n-type)
			9.62 (p-type)	0.36 (p-type)

In this work, we report ultrahigh thermopowers and thermally induced polarization switching from n-type to p-type for the ternary electrolytes consisting of I^−^/I_3_^−^ redox couple, MC, and KCl. Benefiting from the gelation of MC and KCl-induced complexation at elevated temperatures, both Δ*S* and Δ*C* of redox couple are increased, leading to the substantially enhanced thermopowers. Remarkably, the polarization of the ternary electrolytes switches from n-type to p-type above a transition temperature due to the reversed reactions at two electrodes induced by the hydrophobic interaction between MC and I_3_^−^ ions at the hot electrode. The optimized electrolyte, I^−^/I_3_^−^ + 2 wt % MC + 0.3 M KCl, achieves record-high thermogalvanic thermopowers of −8.18 mV K^−1^ for n-type and 9.62 mV K^−1^ for p-type. In addition, KCl effectively lowers the transition temperature of polarization switching. With the optimized ternary electrolyte, our single n-type and p-type TGCs give rise to maximum output powers of 27.78 and 80.47 μW, respectively, under Δ*T* = 15°C. The normalized maximum power densities of our n-type and p-type TGCs are 0.12 and 0.36 mW m^−2^ K^−2^, respectively, which outperform other I^−^/I_3_^−^-based TGCs reported in the literature. The polarized electrolyte reported in this work enables high-performance and cost-effective liquid thermocells for low-grade heat harvesting.

## MATERIALS AND METHODS

### Materials

Potassium iodide (KI), iodine (I_2_), KCl, and MC were purchased from Dieckmann (Hong Kong) Chemical Industry Co. Ltd. and used without further purification. Graphite electrodes, copper plates, and Teflon frame were purchased from Shenzhen SOGAA Technology Co. Ltd.

### Electrolyte preparation

The pristine I^−^/I_3_^−^ electrolyte was prepared by dissolving 5 mmol of KI and 2.5 mmol of I_2_ into 0.5 liters of deionized water with the aid of ultrasonication. To prepare the I^−^/I_3_^−^ + *x* MC electrolyte, 15 ml of pristine I^−^/I_3_^−^ electrolyte was heated up to 70°C in a water bath. Then, a certain amount of dry MC powders, corresponding to *x* wt %, was added into the preheated solution. The solution was subject to magnetic stirring for half an hour until the dispersion became homogeneous. The ternary electrolyte was prepared on the basis of the I^−^/I_3_^−^ + 2 wt % MC electrolyte by adding various contents of KCl from 0.1 to 0.8 M. The obtained electrolytes were stored in a refrigerator at ~2°C for at least 48 hours before further usage.

### Electrolyte characterization

The UV-Vis spectroscopy characterization (LabRAM, HR800) was conducted to determine the relative concentration changes of I^−^ and I_3_^−^ ions at hot and cold electrodes. The pristine I^−^/I_3_^−^ electrolyte was characterized as a reference. For the TGCs with the binary and ternary electrolytes, the samples were taken near two electrodes. To make comparison, the same amount of sample was taken, filtered, and diluted 20 times for each UV-Vis measurement. The I^−^/I_3_^−^ absorbance intensity of the pristine electrolyte is regarded as the reference intensity (*I*_0_). The relative concentration changes of I^−^ and I_3_^−^ ions are determined from the change in the absorbance intensity (Δ*I*) by Δ*C*/*C*_0_ = Δ*I*/*I*_0_. The CV measurements were performed using a three-electrode setup with a potentiostat/galvanostat (Princeton Applied Research, VersaSTAT 3F) as shown in Fig. 4A. As the developed electrolytes are temperature sensitive, the CV curves were obtained at different temperatures with a scanning rate of 10 mV/s. The FTIR spectroscopy characterization (Bruker, Alpha) was conducted to examine the interactions between MC and K^+^/I_3_^−^ ions. The electrolyte was dried overnight under vacuum at 60°C. The obtained solid was used for the FTIR characterization. The micro-DSC measurements (PerkinElmer) were conducted to determine the gelation temperatures of pure MC and dry powders of the I^−^/I_3_^−^ + 2 wt % MC + *y* KCl electrolytes (*y* = 0.1, 0.2, 0.3, 0.5, and 0.8 M). The samples were prepared by drying the electrolytes overnight until the dry powders were obtained. The ionic conductivity of the electrolyte was measured with a conductivity meter (Eutech PC 700) from 25° to 70°C via the direct current (DC) method. The XRD characterization (Rigaku, SmartLab) was performed on the pure MC and 2 wt % MC + 0.3 M KCl powders to examine the materials crystallinity. The 2 wt % MC + 0.3 M KCl powder was obtained by vacuum drying the aqueous mixture.

### Characterization of the thermopower and power generation performance

The thermopower of the prepared electrolytes and power generation performance of single TGCs were characterized by using a homemade setup (fig. S2). The cell consists of a Teflon frame, symmetric graphite electrodes, and copper plates as current collectors. Before testing, the electrolyte was filled in the cavity. Thermocouples were installed at the inner surfaces of graphite electrodes to monitor the electrode temperature. For characterizing the thermopower, the cold electrode is held at 24°C, while the hot electrode is heated up slowly. The electrode temperatures and the generated open-circuit voltage were recorded simultaneously with two data loggers (Agilent, 34970A). For characterizing the output power, a potentiometer is connected with a TGC and serves as the external load. During the testing, the temperature difference between two electrodes is held at 5°C or 15°C. The load voltage and current were recorded with a data logger (Agilent, 34970A) and a source meter (Keithley, 2425), respectively, when gradually tuning the load resistance.

## Supplementary Materials

### This PDF file includes:

Note S1 Figs. S1 to S15 Table S1 References

## References

[1] C. Forman, I. K. Muritala, R. Pardemann, B. Meyer, Estimating the global waste heat potential. Renew. Sustain. Energy Rev. 57, 1568–1579 (2016).

[2] M. F. Dupont, D. R. MacFarlane, J. M. Pringle, Thermo-electrochemical cells for waste heat harvesting-progress and perspectives. Chem. Commun. 53, 6288–6302 (2017). 10.1039/c7cc02160g 28534592

[3] R. Hu, D. Xu, X. Luo, Liquid thermocells enable low-grade heat harvesting. Matter 3, 1400–1402 (2020).

[4] C. Cheng, Y. Dai, J. Yu, C. Liu, S. Wang, S. P. Feng, M. Ni, Review of liquid-based systems to recover low-grade waste heat for electrical energy generation. Energy Fuel 35, 161–175 (2021).

[5] J. Duan, B. Yu, L. Huang, B. Hu, M. Xu, G. Feng, J. Zhou, Liquid-state thermocells: Opportunities and challenges for low-grade heat harvesting. Joule 5, 768–779 (2021).

[6] D. Zhao, H. Wang, Z. U. Khan, J. Chen, R. Gabrielsson, M. P. Jonsson, M. Berggren, X. Crispin, Ionic thermoelectric supercapacitors. Energ. Environ. Sci. 9, 1450–1457 (2016).

[7] D. Zhao, A. Martinelli, A. Willfahrt, T. Fischer, D. Bernin, Z. U. Khan, M. Shahi, J. Brill, M. P. Jonsson, S. Fabiano, X. Crispin, Polymer gels with tunable ionic Seebeck coefficient for ultra-sensitive printed thermopiles. Nat. Commun. 10, 1093 (2019). 30842422 10.1038/s41467-019-08930-7 PMC6403253

[8] T. I. Quickenden, C. F. Vernon, Thermogalvanic conversion of heat to electricity. Sol. Energy 36, 63–72 (1986).

[9] T. I. Quickenden, Y. Mua, A review of power generation in aqueous thermogalvanic cells. J. Electrochem. Soc. 142, 3985–3994 (1995).

[10] H. J. V. Tyrrell, D. A. Taylor, C. M. Williams, The ‘Seebeck effect’ in a purely ionic system. Nature 177, 668–669 (1956). 10.1038/177668b0 13321934

[11] T. Li, X. Zhang, S. D. Lacey, R. Mi, X. Zhao, F. Jiang, J. Song, Z. Liu, G. Chen, J. Dai, Y. Yao, S. Das, R. Yang, R. M. Briber, L. Hu, Cellulose ionic conductors with high differential thermal voltage for low-grade heat harvesting. Nat. Mater. 18, 608–613 (2019). 30911121 10.1038/s41563-019-0315-6

[12] M. Bonetti, S. Nakamae, M. Roger, P. Guenon, Huge Seebeck coefficients in nonaqueous electrolytes. J. Chem. Phys. 134, 114513 (2011). 21428638 10.1063/1.3561735

[13] T. Kim, J. S. Lee, G. Lee, H. Yoon, J. Yoon, T. J. Kang, Y. H. Kim, High thermopower of ferri/ferrocyanide redox couple in organic-water solutions. Nano Energy 31, 160–167 (2017).

[14] L. Jin, G. W. Greene, D. R. MacFarlane, J. M. Pringle, Redox-active quasi-solid-state electrolytes for thermal energy harvesting. ACS Energy Lett. 1, 654–658 (2016).

[15] T. J. Abraham, D. R. MacFarlane, J. M. Pringle, High Seebeck coefficient redox ionic liquid electrolytes for thermal energy harvesting. Energ. Environ. Sci. 6, 2639–2645 (2013).

[16] T. J. Abraham, D. R. MacFarlane, R. H. Baughman, L. Jin, N. Li, J. M. Pringle, Towards ionic liquid-based thermoelectrochemical cells for the harvesting of thermal energy. Electrochim. Acta 113, 87–93 (2013).

[17] T. J. Abraham, D. R. MacFarlane, J. M. Pringle, Seebeck coefficients in ionic liquids-prospects for thermo-electrochemical cells. Chem. Commun. 47, 6260–6262 (2011). 10.1039/c1cc11501d 21544302

[18] H. Zhou, T. Yamada, N. Kimizuka, Supramolecular thermo-electrochemical cells: Enhanced thermoelectric performance by host-guest complexation and salt-induced crystallization. J. Am. Chem. Soc. 138, 10502–10507 (2016). 27508406 10.1021/jacs.6b04923

[19] J. Duan, B. Yu, K. Liu, J. Li, P. Yang, W. Xie, G. Xue, R. Liu, H. Wang, J. Zhou, P-N conversion in thermogalvanic cells induced by thermo-sensitive nanogels for body heat harvesting. Nano Energy 57, 473–479 (2019).

[20] H. Zhou, T. Yamada, N. Kimizuka, Thermo-electrochemical cells empowered by selective inclusion of redox-active ions by polysaccharides. Sustain. Energy Fuels 2, 472–478 (2018).

[21] S. Sahami, M. J. Weaver, Entropic and enthalpic contributions to the solvent dependence of the thermodynamics of transition-metal redox couples: Part II. Couples containing ammine and ethylenediamine ligands. J. Electroanal. Chem. Interfacial Electrochem. 122, 171–181 (1981).

[22] J. Duan, G. Feng, B. Yu, J. Li, M. Chen, P. Yang, J. Feng, K. Liu, J. Zhou, Aqueous thermogalvanic cells with a high Seebeck coefficient for low-grade heat harvest. Nat. Commun. 9, 5146 (2018). 30514952 10.1038/s41467-018-07625-9 PMC6279834

[23] B. Yu, J. Duan, H. Cong, W. Xie, R. Liu, X. Zhuang, H. Wang, B. Qi, M. Xu, Z. Wang, J. Zhou, Thermosensitive crystallization-boosted liquid thermocells for low-grade heat harvesting. Science 370, 342–346 (2020). 32913001 10.1126/science.abd6749

[24] C. Han, X. Qian, Q. Li, B. Deng, Y. Zhao, Z. Han, W. Zhang, W. Wang, S. P. Feng, G. Chen, W. Liu, Giant thermopower of ionic gelatin near room temperature. Science 368, 1091–1098 (2020). 32354840 10.1126/science.aaz5045

[25] M. C. Tate, D. A. Shear, S. W. Hoffman, D. G. Stein, M. C. LaPlaca, Biocompatibility of methylcellulose-based constructs designed for intracerebral gelation following experimental traumatic brain injury. Biomaterials 22, 1113–1123 (2001). 11352091 10.1016/s0142-9612(00)00348-3

[26] K. Nishinari, K. E. Hofmann, H. Moritaka, K. Kohyama, N. Nishinari, Gel-sol transition of methylcellulose. Macromol. Chem. Phys. 198, 1217–1226 (1997).

[27] K. Kobayashi, C. I. Huang, T. P. Lodge, Thermoreversible gelation of aqueous methylcellulose solutions. Macromolecules 32, 7070–7077 (1999).

[28] J. W. Minns, A. Khan, α-Cyclodextrin-I_3_^−^ host-guest complex in aqueous solution: Theoretical and experimental studies. J. Phys. Chem. A 106, 6421–6425 (2002).

[29] Y. Huang, W. Guo, J. Zhang, X. Peng, G. Li, L. M. Zhang, L. Yang, Thermosensitive hydrogels based on methylcellulose derivatives for prevention of postoperative adhesion. Cellulose 27, 1555–1571 (2020).

[30] Q. Fu, Y. C. Xiong, W. H. Zhang, D. Y. Xu, A setup for measuring the Seebeck coefficient and the electrical resistivity of bulk thermoelectric materials. Rev. Sci. Instrum. 88, 095111 (2017). 28964241 10.1063/1.4990634

[31] P. P. Kundu, M. Kundu, Effect of salts and surfactant and their doses on the gelation of extremely dilute solutions of methyl cellulose. Polymer 42, 2015–2020 (2001).

[32] A. A. Zavitsas, Properties of water solutions of electrolytes and nonelectrolytes. J. Phys. Chem. B 105, 7805–7817 (2001).

[33] Y. Xu, C. Wang, K. C. Tam, L. Li, Salt-assisted and salt-suppressed sol-gel transitions of methylcellulose in water. Langmuir 20, 646–652 (2004). 15773087 10.1021/la0356295

[34] R. H. Gerke, Temperature coefficient of electromotive force of galvanic cells and the entropy of reactions. J. Am. Chem. Soc. 44, 1684–1704 (1922).

[35] N. E. A. Shuhaimi, L. P. Teo, H. J. Woo, S. R. Majid, A. K. Arof, Electrical double-layer capacitors with plasticized polymer electrolyte based on methyl cellulose. Polym. Bull. 69, 807–826 (2012).

[36] N. E. A. Shuhaimi, S. R. Majid, A. K. Arof, On complexation between methyl cellulose and ammonium nitrate. Mater. Res. Innov. 13, 239–242 (2009).

[37] L. Zhang, T. Kim, N. Li, T. J. Kang, J. Chen, J. M. Pringle, M. Zhang, A. H. Kazim, S. Fang, C. Haines, D. Al-Masri, B. A. Cola, J. M. Razal, J. Di, S. Beirne, D. R. MacFarlane, A. Gonzalez-Martin, S. Mathew, Y. H. Kim, G. Wallace, R. H. Baughman, High power density electrochemical thermocells for inexpensively harvesting low-grade thermal energy. Adv. Mater. 29, 1605652 (2017). 10.1002/adma.201605652 28121372

[38] H. Im, T. Kim, H. Song, J. Choi, J. S. Park, R. Ovalle-Robles, H. Yang, K. D. Kihm, R. H. Baughman, H. H. Lee, High-efficiency electrochemical thermal energy harvester using carbon nanotube aerogel sheet electrodes. Nat. Commun. 7, 10600 (2016). 26837457 10.1038/ncomms10600 PMC4742963

[39] R. Hu, B. A. Cola, N. Haram, J. N. Barisci, S. Lee, S. Stoughton, G. Wallace, C. Too, M. Thomas, A. Gestos, M. E. Cruz, J. P. Ferraris, A. A. Zakhidov, R. H. Baughman, Harvesting waste thermal energy using a carbon-nanotube-based thermo-electrochemical cell. Nano Lett. 10, 838–846 (2010). 20170193 10.1021/nl903267n

[40] A. J. deBethume, T. S. Licht, N. Swendeman, The temperature coefficients of electrode potentials: The isothermal and thermal coefficients—The standard ionic entropy of the electrochemical transport of the hydrogen ion. J. Electrochem. Soc. 106, 616–625 (1959).

[41] Z. Karpas, Z. Berant, O. Shahal, Effect of temperature on the mobility of ions. J. Am. Chem. Soc. 111, 6015–6018 (1989).

