# Supplementary material for: High-thermopower polarized electrolytes enabled by methylcellulose for low-grade heat harvesting
Source: Sci Adv. 2022 Feb 18;8(7):eabl5318. doi: 10.1126/sciadv.abl5318 (PMC8856612; doi:10.1126/sciadv.abl5318)
Supplement: Supplementary file 1 — Note S1 Figs. S1 to S15 Table S1 References [file sciadv.abl5318_sm.pdf]

Science Advances
AAAS

Supplementary Materials for

High-thermopower polarized electrolytes enabled by methylcellulose for low-grade heat harvesting

Yang Han, Jian Zhang, Run Hu*, Dongyan Xu*

*Corresponding author. Email: hurun@hust.edu.cn (R.H.); dyxu@mae.cuhk.edu.hk (D.X.)

Published 18 February 2022, *Sci. Adv.* **8**, eabl5318 (2022)
DOI: 10.1126/sciadv.abl5318

This PDF file includes:

Note S1
Figs. S1 to S15
Table S1
References

Note S1. Thermopower of a redox couple

According to the Nernst equation, for an electrochemical reaction $A\ ox + n\ \mathrm{e} \rightleftarrows B\ red$, the equilibrium potential E can be expressed as:

$$E = E^0 + \frac{RT}{nF}\ln\frac{\left(a_{ox}\right)^A}{\left(a_{red}\right)^B} \tag{1}$$

where E^0 is the standard potential, n is the number of electrons transferred in the electrochemical reaction, F is the Faraday constant, R is the ideal gas constant, the exponents A and B are the coefficients in the reaction, a is the activity of oxidation or reduction species as indicated by the subscript ox or red. a is related to the concentration (C) by $a = \gamma C$, where γ is the activity coefficient. Thus, Eq. (1) can be rewritten as:

$$E = E^0 + \frac{RT}{nF}\left[\ln\frac{\left(\gamma_{ox}\right)^A}{\left(\gamma_{red}\right)^B} + \ln\frac{\left(C_{ox}\right)^A}{\left(C_{red}\right)^B}\right] \tag{2}$$

The thermopower (S_{TGC}) of a redox couple can be defined as

$$S_{TGC} = -\frac{E_H - E_C}{T_H - T_C} \tag{3}$$

where E and T are the equilibrium potential and temperature of the electrode, the subscripts H and C denote hot and cold electrodes. It is worth noting that the definition of temperature coefficient in electrochemistry has opposite sign convention from Seebeck coefficient in thermoelectrics (*4*). Recently, Han et al. gave a thorough discussion on the sign conventions of thermogalvanic and thermodiffusion thermopowers and they followed the sign convention of Seebeck coefficient for the definition of thermopower (*24*). We follow the same sign convention in this work. By substituting Eq. (2) into Eq. (3), we obtain

$$S_{TGC} = -\frac{E_H^0 - E_C^0}{T_H - T_C} - \frac{R}{nF\Delta T}\left[T_H\ln\frac{\left(\gamma_{ox,H}\right)^A}{\left(\gamma_{red,H}\right)^B} - T_C\ln\frac{\left(\gamma_{ox,C}\right)^A}{\left(\gamma_{red,C}\right)^B}\right] - \frac{R}{nF\Delta T}\left[T_H\ln\frac{\left(C_{ox,H}\right)^A}{\left(C_{red,H}\right)^B} - T_C\ln\frac{\left(C_{ox,C}\right)^A}{\left(C_{red,C}\right)^B}\right] \tag{4}$$

In electrochemistry, the temperature coefficient (α_R) is defined as (*40*)

$$\alpha_R = \frac{E_H^0 - E_C^0}{T_H - T_C} \tag{5}$$

α_R can be further related to the entropy change of redox reaction as follows:

$$\alpha_R = \frac{S_{red} - S_{ox}}{nF} = \frac{\Delta S}{nF} \tag{6}$$

where S_{red} and S_{ox} are the partial molar entropies of reduction and oxidation species, and ΔS is the partial molar entropy change. Substituting Eqs. (5) and (6) into Eq. (4), we obtain

$$S_{TGC} = -\frac{\Delta S}{nF} - \frac{R}{nF\Delta T}\left[T_H\ln\frac{\left(\gamma_{ox,H}\right)^A}{\left(\gamma_{red,H}\right)^B} - T_C\ln\frac{\left(\gamma_{ox,C}\right)^A}{\left(\gamma_{red,C}\right)^B}\right] - \frac{R}{nF\Delta T}\left[T_H\ln\frac{\left(C_{ox,H}\right)^A}{\left(C_{red,H}\right)^B} - T_C\ln\frac{\left(C_{ox,C}\right)^A}{\left(C_{red,C}\right)^B}\right] \tag{7}$$

The second term on the right-hand side of Eq. (7) is related to the entropy change of redox species due to other activities such as the phase transition of MC and KCl-induced complexation/dissociation. The third term is related to the concentration difference of redox species between two electrodes.

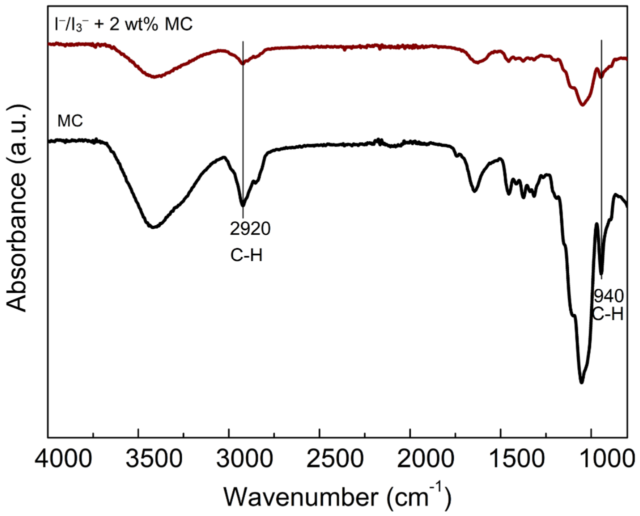

Fig. S1. FTIR characterization of the hydrophobic interaction between MC and I_3^- ions. FTIR spectra of the pure MC and I^-/I_3^- + 2 wt% MC powders.

Fig. S1 shows the FTIR spectra of the pure MC and I^-/I_3^- + 2 wt% MC powders. Several absorption peaks corresponding to O-H stretching (3414 cm^{-1}), C-H stretching (2920 and 940 cm^{-1}), C-O-C stretching (1635 cm^{-1}) on the glucose ring, and $-OCH_3$ groups (1048 cm^{-1}) are observed for the pure MC. For the I^-/I_3^- + 2 wt% MC powders, C-H stretching is greatly weakened, indicating that I_3^- ions interact with MC through the hydrophobic methyl groups.

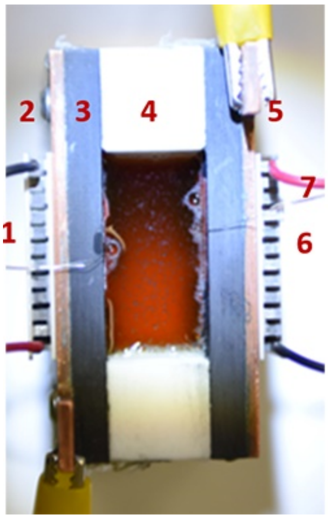

Fig. S2. Experimental setup for characterizing the thermopower and power generation performance. Parts of the experimental setup: 1, 6 – thermoelectric devices for controlling the temperatures of hot and cold electrodes; 2, 5 – copper plates as current collectors; 3 – graphite plates as the electrodes; 4 – Teflon frame with a thickness of 1.5 cm for thermal and electrical insulation; 7 – thermocouples installed at the inner surface of the electrodes for monitoring the temperature.

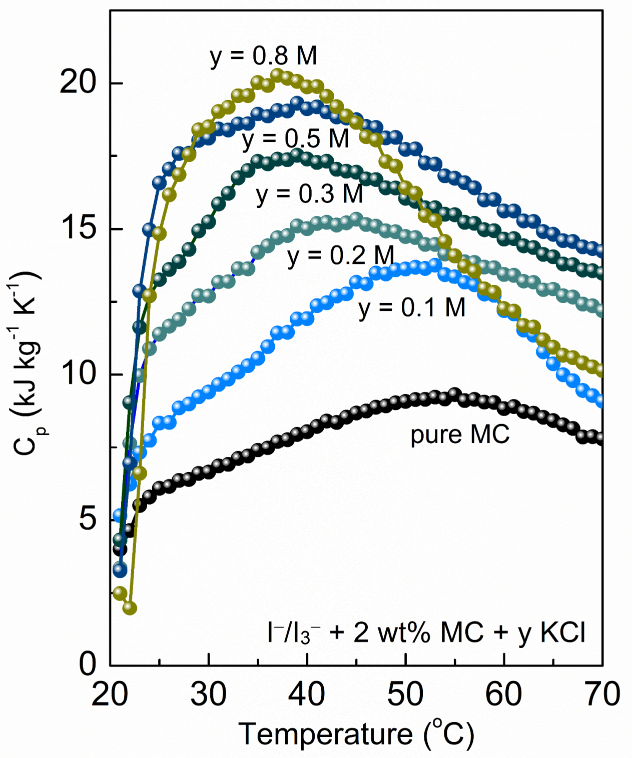

Fig. S3. Micro DSC characterization of pure MC and ternary electrolytes. Measured specific heat (C_p) as a function of temperature for pure MC and dry powders of the I^-/I_3^- + 2 wt% MC + y KCl electrolytes (y = 0.1, 0.2, 0.3, 0.5, and 0.8 M).

The micro differential scanning calorimetry (DSC) measurements were conducted to determine the gelation temperatures of pure MC and dry powders of the I^-/I_3^- + 2 wt% MC + y KCl electrolytes (y = 0.1, 0.2, 0.3, 0.5, and 0.8 M). Fig. S3 shows the measured specific heat as a function of temperature for each sample. An endothermic peak is observed, which is due to the sol-gel transition of MC. The gelation temperature can be determined as the temperature corresponding to the peak specific heat. The extracted gelation temperatures of the ternary electrolytes are listed in Table 1, which agree reasonably well with the n-p transition temperatures, confirming that polarization switching is induced by the gelation of MC. As shown in Fig. S3, KCl effectively lowers the gelation temperature of MC. The area under the heat capacity curve represents the enthalpy change during the sol-gel process. Compared to the pure MC, the addition of KCl leads to a larger enthalpy change, indicating an increase in the entropy change.

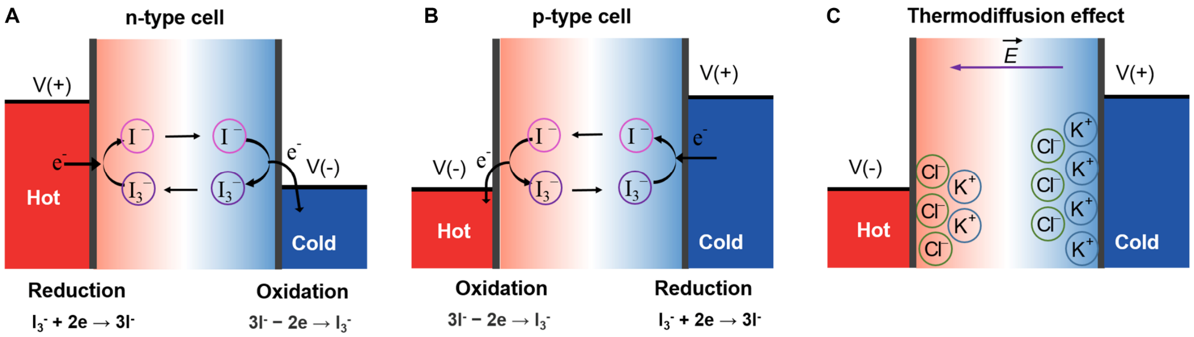

Fig. S4. Schematics of thermogalvanic and thermodiffusion effects. (A) n-type thermogalvanic effect; (B) p-type thermogalvanic effect; (C) thermodiffusion effect.

For the pristine I^-/I_3^- electrolyte, reduction will occur at the hot electrode (Fig. S4A). Thus, electrons will flow out of the hot electrode and into the cold electrode, which is equivalent to n-type materials in thermoelectrics. In this work, the thermopower is defined as $S_{TGC} = -\Delta V/\Delta T$. The n-type electrolyte has a negative thermopower, which is consistent with the sign convention in thermoelectrics.

When the hot electrode temperature is below the transition temperature, the MC-added I^-/I_3^- electrolyte behaves as n-type. When the hot electrode temperature is higher than the transition temperature, the reversed reactions will occur at two electrodes induced by the strong hydrophobic interaction between MC and I_3^- ions at the hot electrode. Then, the electrolyte turns into p-type, as shown in Fig. S4B. For the ternary electrolytes, 0.1 – 0.8 M KCl was added to enhance the thermopower and tune the transition temperature for polarization switching. The thermodiffusion effect of mobile ions will also contribute to the thermopower, as shown in Fig. S4C.

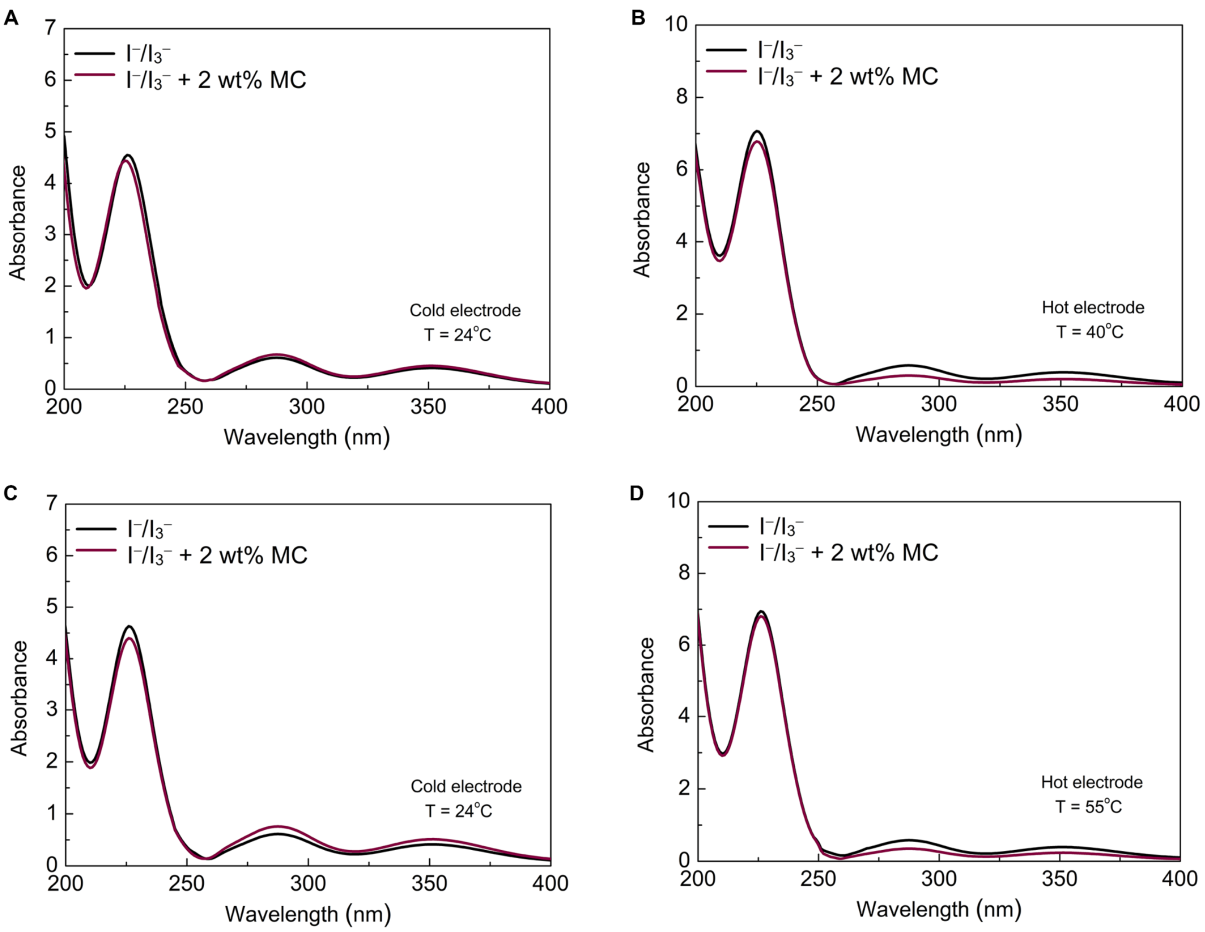

Fig. S5. UV-Vis spectra of the pristine I^-/I_3^- and I^-/I_3^- + 2 wt% MC electrolytes. UV-Vis spectra at two electrodes for the TGCs with a hot electrode temperature of (A, B) 40°C and (C, D) 55°C. The UV-Vis spectra of the pristine I^-/I_3^- electrolyte are also shown in (A-D) as a reference.

The UV-Vis spectra in the wavelength range of 200 – 400 nm are shown in Fig. S5 for both the pristine I^-/I_3^- and I^-/I_3^- + 2 wt% MC electrolytes. The absorption peak near 225 nm is ascribed to I^- ions, while those peaks at 290 and 350 nm are ascribed to I_3^- ions. The absorbance profile of the pristine I^-/I_3^- electrolyte serves as the reference. Generally, the I^- concentration of the I^-/I_3^- + 2 wt% MC electrolyte at both electrodes is nearly the same as that of the pristine I^-/I_3^- electrolyte, indicating that I^- ions do not involve in complexation. In contrast, the I_3^- concentration decreases at the hot electrode with the increase of temperature, while it increases at the cold electrode accordingly. This confirms that there is the strong hydrophobic interaction between MC and I_3^-ions at the hot electrode and the interaction is more intense at higher temperatures.

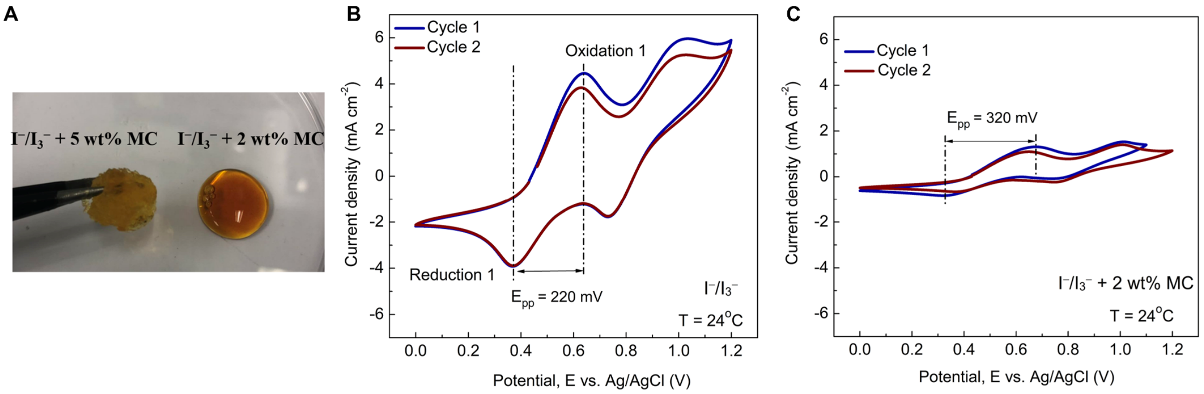

Fig. S6. Effects of MC on the I^-/I_3^- electrolyte. (A) Photograph of the I^-/I_3^- + x MC electrolytes with x = 2 and 5 wt%, respectively. CV curves of (B) the pristine I^-/I_3^- and (C) I^-/I_3^- + 2 wt% MC electrolytes at room temperature.

Fig. S6A clearly shows that a high MC content (5 wt%) will solidify the electrolyte while the I^-/I_3^- + 2 wt% MC electrolyte remains as solution at room temperature. As a nonconductive polymer, MC may hinder the electrochemical activities of the electrolyte. The CV measurements were conducted on both the pristine I^-/I_3^- and I^-/I_3^- + 2 wt% MC electrolytes in a three-electrode configuration at room temperature. As seen in Fig. S6B and C, both electrolytes undergo a two-step oxidation process. The peak separation (E_{pp}) of a CV curve, which is the potential difference between oxidation 1 and reduction 1 peaks, reflects the reversibility of the electrochemical reaction. Compared to the pristine electrolyte (E_{pp} = 220 mV), the E_{pp} of the I^-/I_3^- + 2 wt% MC electrolyte (320 mV) is larger, indicating the degraded reversibility.

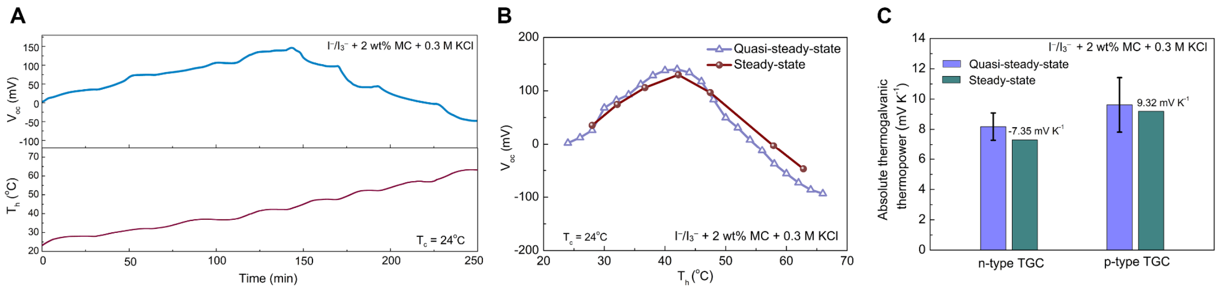

Fig. S7. Steady-state thermopower measurement and comparison of the thermopowers extracted by the quasi-steady-state differential method and the steady-state method. (A) Recorded open-circuit voltage and hot electrode temperature over time for the I^-/I_3^- + 2 wt% MC + 0.3 M KCl electrolyte during the steady-state measurement. (B) Comparison of the V_{oc}-T_h curves obtained by the quasi-steady-state differential method and the steady-state method. (C) Comparison of the absolute thermopowers obtained by two methods.

Fig. S7A shows the recorded open-circuit voltage and hot electrode temperature over time during the steady-state measurement. Fig. S7B compares the V_{oc}-T_h curves obtained by the quasi-steady-state differential method and the steady-state method, which agree with each other reasonably well. As shown in Fig. S7C, the thermopowers obtained by the steady-state method are −7.35 mV K^{-1} for n-type and 9.32 mV K^{-1} for p-type, both of which are consistent with the mean thermopowers extracted by the quasi-steady-state differential method within the error bar.

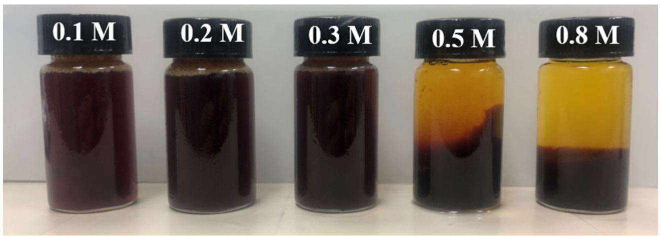

Fig. S8. Photograph of the I^-/I_3^- + 2 wt% MC + y KCl ternary electrolytes (y = 0.1, 0.2, 0.3, 0.5, and 0.8 M).

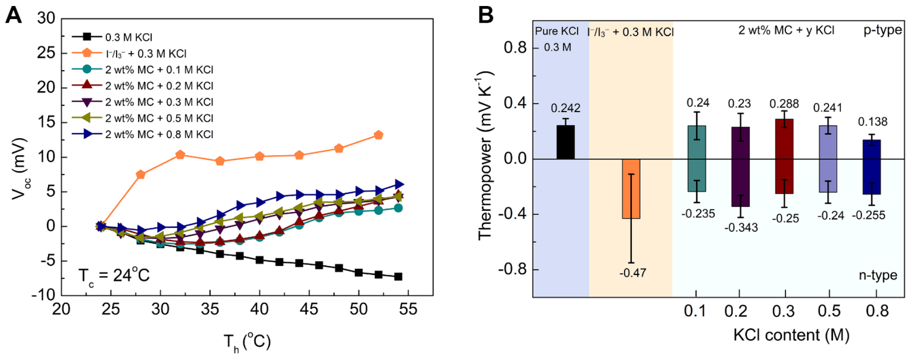

Fig. S9. Thermopowers of the pure 0.3 M KCl, I^-/I_3^- + 0.3 M KCl, and 2 wt% MC + y KCl electrolytes (y = 0.1, 0.2, 0.3, 0.5, and 0.8 M). (A) Recorded open-circuit voltage (V_{oc}) with the increase of the hot electrode temperature for the pure 0.3 M KCl, I^-/I_3^- + 0.3 M KCl, and 2 wt% MC + y KCl electrolytes (y = 0.1, 0.2, 0.3, 0.5, and 0.8 M). The cold electrode temperature is held at 24°C. (B) Extracted thermopowers.

In addition to the thermogalvanic effect of the I^-/I_3^- redox couple, the thermodiffusion effect of mobile ions in the ternary electrolytes will also contribute to the thermopower. Therefore, we further characterized the thermodiffusion thermopowers of the pure 0.3 M KCl and 2 wt% MC + y KCl electrolytes, as shown in Fig. S9. For the pure 0.3 M KCl electrolyte, the open-circuit voltage drops as temperature increases, indicating a positive thermopower. As shown in Fig. S4C, under a temperature gradient, both K^+ and Cl^- ions diffuse from the hot electrode to the cold one. Due to their different thermal diffusivities, more K^+ ions will accumulate at the cold electrode, which will lead to a p-type cell. However, for the 2 wt% MC + y KCl electrolytes, polarization switching from p-type to n-type occurs above a certain transition temperature, as suggested by the first decreasing and then increasing temperature trend of the recorded open-circuit voltages. MC interacts with both K^+ and Cl^- ions. Upon heating, MC gradually turns into gelation at the hot electrode and will restrict the ion mobility. As a result, K^+ and Cl^- ions diffuse back from the cold electrode to the hot one. More K^+ ions accumulate at the hot electrode instead, leading to an n-type cell. Fig. S9B shows that the absolute thermodiffusion thermopowers of the 2 wt% MC + y KCl electrolytes are less than 0.4 mV K^{-1}, which are much lower than the absolute thermopowers of the optimized ternary electrolyte (8.18 mV K^{-1} for n-type and 9.62 mV K^{-1} for p-type).

The thermopower of the I^-/I_3^- + 0.3 M KCl electrolytes was also characterized for comparison. For the I^-/I_3^- + 0.3 M KCl electrolyte, the V_{oc}-T_h curve is not smooth presumably due to the competition between the n-type thermogalvanic effect of the I^-/I_3^- redox couple and the p-type thermodiffusion effect of KCl. The extracted thermopower is –0.47±0.32 mV K^{-1}, which is slightly lower than the thermopower of the pristine I^-/I_3^- electrolyte (–0.71 mV K^{-1}).

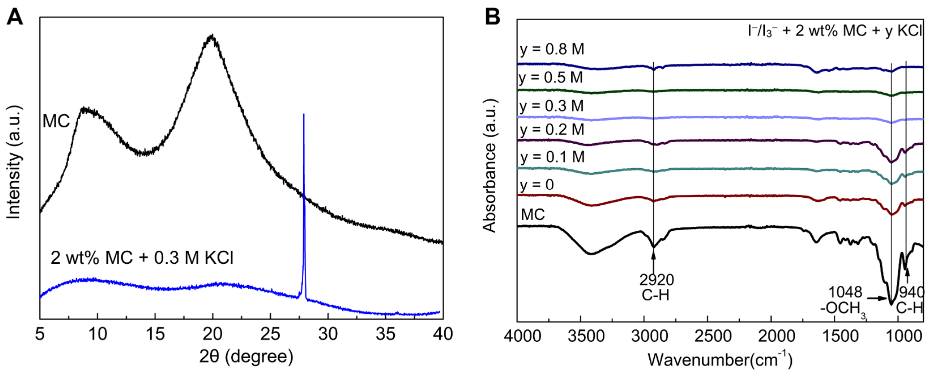

Fig. S10. Characterization of the interaction between MC and K^+ ions. (A) XRD spectra of the pure MC and dried powders of the 2 wt% MC + 0.3 M KCl electrolyte; (B) FTIR spectra of the pure MC and dried powders of the I^-/I_3^- + 2 wt% MC + y KCl electrolytes (y = 0, 0.1, 0.2, 0.3, 0.5, and 0.8 M).

The XRD characterization was performed on the pure MC and dried powders of the 2 wt% MC + 0.3 M KCl electrolyte to investigate the interaction between MC and KCl (Fig. S10A). Two relatively broad peaks were observed at around 8.0° and 20.5° for the pure MC, indicating a semi-crystalline structure. Both peaks are weakened dramatically after complexing with KCl, which suggests that MC becomes more amorphous. The FTIR spectra of the I^-/I_3^- + 2 wt% MC + y KCl electrolytes in Fig. S10B shows that the absorption peak at 1048 cm^{-1} for $–OCH_3$ stretching mode broadens, confirming that K^+ ions interact with MC at O sites.

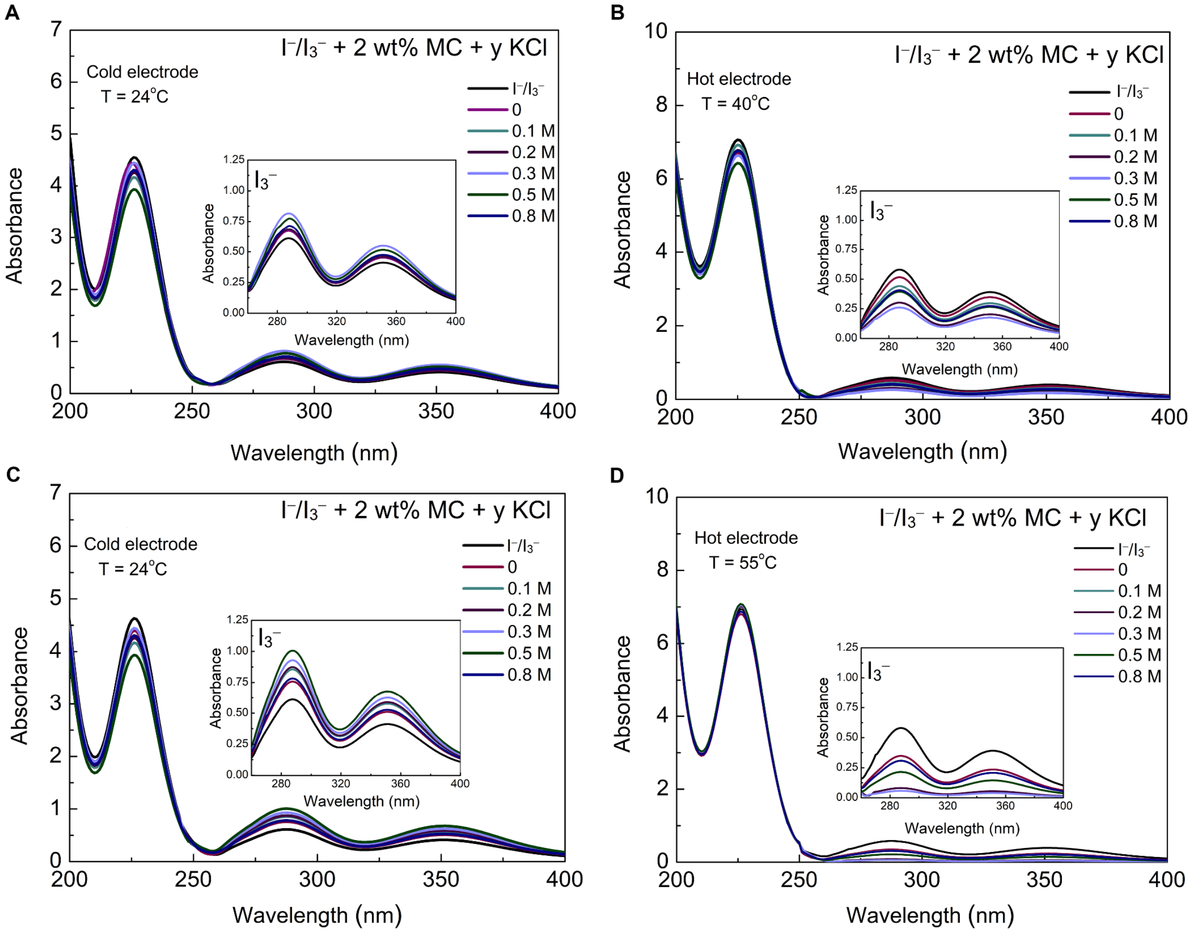

Fig. S11. UV-Vis spectra of the pristine I^-/I_3^- and I^-/I_3^- + 2 wt% MC + y KCl electrolytes (y = 0, 0.1, 0.2, 0.3, 0.5, and 0.8 M). UV-Vis spectra at two electrodes for the TGCs with a hot electrode temperature of (A, B) 40°C and (C, D) 55°C. The UV-Vis spectrum of the pristine I^-/I_3^- electrolyte is also shown in (A-D) as a reference. (Insets show the zoomed in absorbance spectra of I_3^- ions)

The UV-Vis spectroscopy characterization was conducted to examine the relative concentration changes of I^- and I_3^- ions at cold and hot electrodes for the I^-/I_3^- + 2 wt% MC + y KCl electrolytes (y = 0, 0.1, 0.2, 0.3, 0.5, and 0.8 M), as shown in Fig. S11. The absorbance profiles of I^- ions nearly overlap with each other, indicating that the I^- concentration is almost unchanged. However, the absorbance of I_3^- ions at the hot electrode first decreases with the increase of the KCl content, reaches a minimum value at 0.3 M, and then increases as the KCl content further rises (Fig. S11B and D). In addition, the TGC with T_h = 55°C demonstrates a larger absorbance change for I_3^- ions than the TGC with T_h = 40°C, which is due to the higher hot electrode temperature and thus the stronger hydrophobicity of MC (Fig. S11B and D). The absorbance profiles of I_3^- ions at the cold electrode show an opposite trend due to the mass transport in a confined cell (Fig. S11A and C). The relative concentration changes ($\Delta C/C_0$) of I^- and I_3^- ions are determined from the corresponding absorbance changes at two electrodes and the results are shown in Fig. 5D-G.

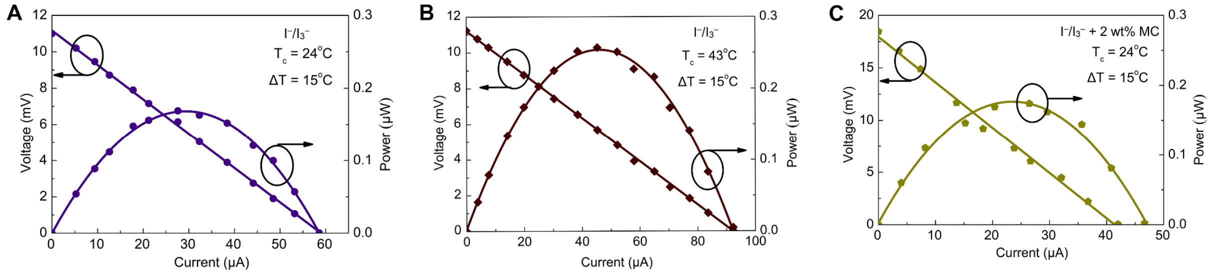

Fig. S12. Power generation performance of the pristine and n-type binary TGCs. Voltage-current curves and output powers of the pristine TGCs with T_c = (A) 24°C and (B) 43°C and (C) n-type TGC with the binary electrolyte.

The voltage-current curves and output powers of the TGCs with the pristine and binary electrolytes are given in Fig. S12. Under ΔT = 15°C, the pristine TGC achieves a maximum power of 0.16 μW and 0.26 μW for T_c = 24°C and 43°C, respectively, while the n-type TGC with the binary electrolyte gives rise to a maximum power of 0.18 μW.

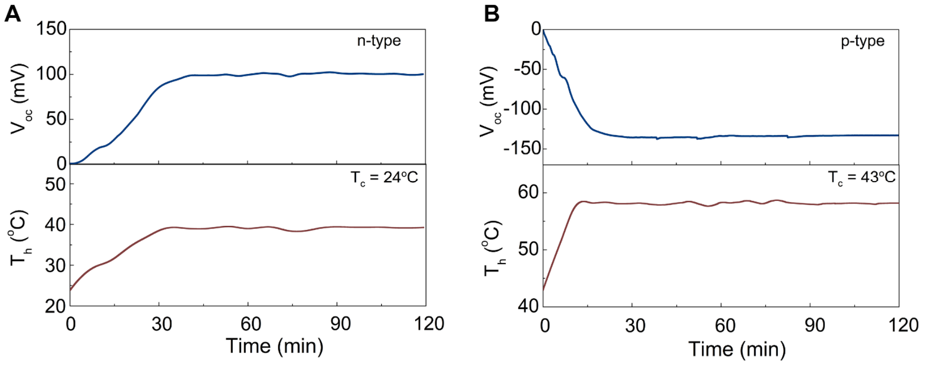

Fig. S13. Durability of n-type and p-type TGCs with the optimized ternary electrolyte. Recorded open-circuit voltage and hot electrode temperature over time for (A) n-type and (B) p-type TGCs with the optimized ternary electrolyte.

The durability of both n-type and p-type TGCs with the optimized ternary electrolyte is examined for a relatively long period (120 min). Fig. S13 shows the recorded open-circuit voltage and hot electrode temperature over time for n-type and p-type TGCs when the hot electrode is gradually heated up till ΔT = 15°C and then maintained at this temperature. It is shown that both n-type and p-type TGCs can maintain a stable output voltage under a constant temperature difference, confirming the durability of our TGCs.

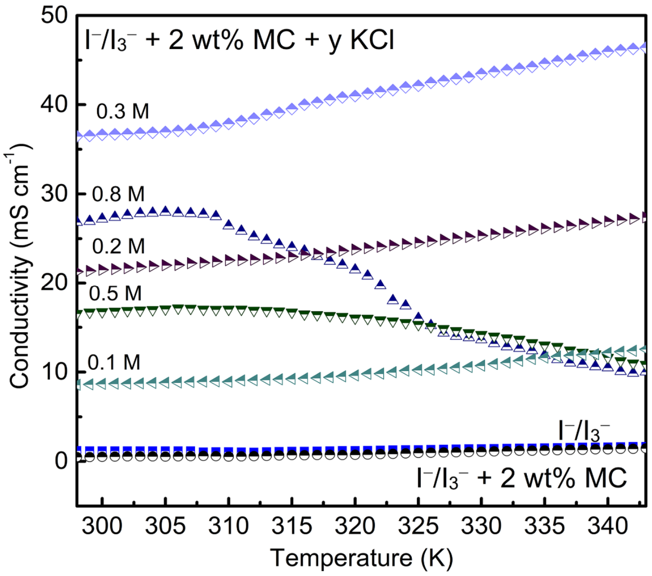

Fig. S14. Ionic conductivity of the pristine, binary, and ternary electrolytes. Temperature-dependent ionic conductivity of the pristine I^-/I_3^-, I^-/I_3^- + 2 wt% MC, and I^-/I_3^- + 2 wt% MC + y KCl electrolytes (y = 0.1, 0.2, 0.3, 0.5, and 0.8 M).

Fig. S14 shows the temperature dependence of the ionic conductivity for the pristine I^-/I_3^-, I^-/I_3^- + 2 wt% MC, and I^-/I_3^- + 2 wt% MC + y KCl electrolytes (y = 0.1, 0.2, 0.3, 0.5, and 0.8 M). For most of the electrolytes (except ternary electrolytes with y = 0.5 and 0.8 M), the ionic conductivity increases with temperature due to the enhanced mobility of mobile ions (*41*). However, for ternary electrolytes with y = 0.5 and 0.8 M, the ionic conductivity first reaches a plateau and then decreases with temperature. Besides, the ionic conductivity of these two electrolytes is lower than that of the ternary electrolyte with 0.3 M KCl. As clearly seen in Fig. S8, the ternary electrolytes with y = 0.5 and 0.8 M are not stable and precipitates are formed at room temperature. With the increase of temperature, the concentration of K^+ ions will be further reduced due to their complexation with MC, which will lead to the decreased ionic conductivity.

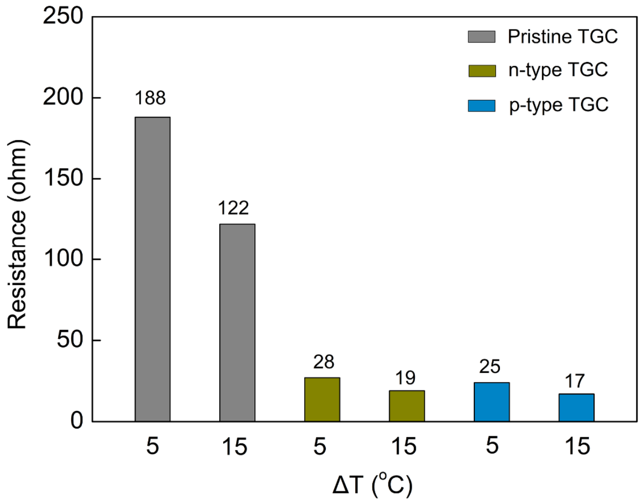

Fig. S15. Internal resistance of the pristine I^-/I_3^- TGC and n-type and p-type TGCs with the optimized ternary electrolyte.

Table S1. Absolute thermogalvanic thermopowers of aqueous electrolytes with different I^-/I_3^- concentrations in the literature.

	**I^-/I_3^- Concentration (mM)**	**Absolute Thermopower (mV K^{-1})**
Zhou[18]	2.5	0.87
Duan[19]	5	0.71
Zhou[20]	12.5	0.86
Zhou[20]	100	0.64
Abraham[17]	400	0.53

REFERENCES AND NOTES

1. C. Forman, I. K. Muritala, R. Pardemann, B. Meyer, Estimating the global waste heat potential. *Renew. Sustain. Energy Rev.* **57**, 1568–1579 (2016).

2. M. F. Dupont, D. R. MacFarlane, J. M. Pringle, Thermo-electrochemical cells for waste heat harvesting-progress and perspectives. *Chem. Commun.* **53**, 6288–6302 (2017).

3. R. Hu, D. Xu, X. Luo, Liquid thermocells enable low-grade heat harvesting. *Matter* **3**, 1400–1402 (2020).

4. C. Cheng, Y. Dai, J. Yu, C. Liu, S. Wang, S. P. Feng, M. Ni, Review of liquid-based systems to recover low-grade waste heat for electrical energy generation. *Energy Fuel* **35**, 161–175 (2021).

5. J. Duan, B. Yu, L. Huang, B. Hu, M. Xu, G. Feng, J. Zhou, Liquid-state thermocells: Opportunities and challenges for low-grade heat harvesting. *Joule* **5**, 768–779 (2021).

6. D. Zhao, H. Wang, Z. U. Khan, J. Chen, R. Gabrielsson, M. P. Jonsson, M. Berggren, X. Crispin, Ionic thermoelectric supercapacitors. *Energ. Environ. Sci.* **9**, 1450–1457 (2016).

7. D. Zhao, A. Martinelli, A. Willfahrt, T. Fischer, D. Bernin, Z. U. Khan, M. Shahi, J. Brill, M. P. Jonsson, S. Fabiano, X. Crispin, Polymer gels with tunable ionic Seebeck coefficient for ultra-sensitive printed thermopiles. *Nat. Commun.* **10**, 1093 (2019).

8. T. I. Quickenden, C. F. Vernon, Thermogalvanic conversion of heat to electricity. *Sol. Energy* **36**, 63–72 (1986).

9. T. I. Quickenden, Y. Mua, A review of power generation in aqueous thermogalvanic cells. *J. Electrochem. Soc.* **142**, 3985–3994 (1995).

10. H. J. V. Tyrrell, D. A. Taylor, C. M. Williams, The ‘Seebeck effect’ in a purely ionic system. *Nature* **177**, 668–669 (1956).

11. T. Li, X. Zhang, S. D. Lacey, R. Mi, X. Zhao, F. Jiang, J. Song, Z. Liu, G. Chen, J. Dai, Y. Yao, S. Das, R. Yang, R. M. Briber, L. Hu, Cellulose ionic conductors with high differential thermal voltage for low-grade heat harvesting. *Nat. Mater.* **18**, 608–613 (2019).

12. M. Bonetti, S. Nakamae, M. Roger, P. Guenon, Huge Seebeck coefficients in nonaqueous electrolytes. *J. Chem. Phys.* **134**, 114513 (2011).

13. T. Kim, J. S. Lee, G. Lee, H. Yoon, J. Yoon, T. J. Kang, Y. H. Kim, High thermopower of ferri/ferrocyanide redox couple in organic-water solutions. *Nano Energy* **31**, 160–167 (2017).

14. L. Jin, G. W. Greene, D. R. MacFarlane, J. M. Pringle, Redox-active quasi-solid-state electrolytes for thermal energy harvesting. *ACS Energy Lett.* **1**, 654–658 (2016).

15. T. J. Abraham, D. R. MacFarlane, J. M. Pringle, High Seebeck coefficient redox ionic liquid electrolytes for thermal energy harvesting. *Energ. Environ. Sci.* **6**, 2639–2645 (2013).

16. T. J. Abraham, D. R. MacFarlane, R. H. Baughman, L. Jin, N. Li, J. M. Pringle, Towards ionic liquid-based thermoelectrochemical cells for the harvesting of thermal energy. *Electrochim. Acta* **113**, 87–93 (2013).

17. T. J. Abraham, D. R. MacFarlane, J. M. Pringle, Seebeck coefficients in ionic liquids-prospects for thermo-electrochemical cells. *Chem. Commun.* **47**, 6260–6262 (2011).

18. H. Zhou, T. Yamada, N. Kimizuka, Supramolecular thermo-electrochemical cells: Enhanced thermoelectric performance by host-guest complexation and salt-induced crystallization. *J. Am. Chem. Soc.* **138**, 10502–10507 (2016).

19. J. Duan, B. Yu, K. Liu, J. Li, P. Yang, W. Xie, G. Xue, R. Liu, H. Wang, J. Zhou, P-N conversion in thermogalvanic cells induced by thermo-sensitive nanogels for body heat harvesting. *Nano Energy* **57**, 473–479 (2019).

20. H. Zhou, T. Yamada, N. Kimizuka, Thermo-electrochemical cells empowered by selective inclusion of redox-active ions by polysaccharides. *Sustain. Energy Fuels* **2**, 472–478 (2018).

21. S. Sahami, M. J. Weaver, Entropic and enthalpic contributions to the solvent dependence of the thermodynamics of transition-metal redox couples: Part II. Couples containing ammine and ethylenediamine ligands. *J. Electroanal. Chem. Interfacial Electrochem.* **122**, 171–181 (1981).

22. J. Duan, G. Feng, B. Yu, J. Li, M. Chen, P. Yang, J. Feng, K. Liu, J. Zhou, Aqueous thermogalvanic cells with a high Seebeck coefficient for low-grade heat harvest. *Nat. Commun.* **9**, 5146 (2018).

23. B. Yu, J. Duan, H. Cong, W. Xie, R. Liu, X. Zhuang, H. Wang, B. Qi, M. Xu, Z. Wang, J. Zhou, Thermosensitive crystallization-boosted liquid thermocells for low-grade heat harvesting. *Science* **370**, 342–346 (2020).

24. C. Han, X. Qian, Q. Li, B. Deng, Y. Zhao, Z. Han, W. Zhang, W. Wang, S. P. Feng, G. Chen, W. Liu, Giant thermopower of ionic gelatin near room temperature. *Science* **368**, 1091–1098 (2020).

25. M. C. Tate, D. A. Shear, S. W. Hoffman, D. G. Stein, M. C. LaPlaca, Biocompatibility of methylcellulose-based constructs designed for intracerebral gelation following experimental traumatic brain injury. *Biomaterials* **22**, 1113–1123 (2001).

26. K. Nishinari, K. E. Hofmann, H. Moritaka, K. Kohyama N. Nishinari, Gel-sol transition of methylcellulose. *Macromol. Chem. Phys.* **198**, 1217–1226 (1997).

27. K. Kobayashi, C. I. Huang T. P. Lodge, Thermoreversible gelation of aqueous methylcellulose solutions. *Macromolecules* **32**, 7070–7077 (1999).

28. J. W. Minns, A. Khan, α-Cyclodextrin-I_3^- host-guest complex in aqueous solution: Theoretical and experimental studies. *J. Phys. Chem. A* **106**, 6421–6425 (2002).

29. Y. Huang, W. Guo, J. Zhang, X. Peng, G. Li, L. M. Zhang, L. Yang, Thermosensitive hydrogels based on methylcellulose derivatives for prevention of postoperative adhesion. *Cellulose* **27**, 1555–1571 (2020).

30. Q. Fu, Y. C. Xiong, W. H. Zhang, D. Y. Xu, A setup for measuring the Seebeck coefficient and the electrical resistivity of bulk thermoelectric materials. *Rev. Sci. Instrum.* **88**, 095111 (2017).

31. P. P. Kundu, M. Kundu, Effect of salts and surfactant and their doses on the gelation of extremely dilute solutions of methyl cellulose. *Polymer* **42**, 2015–2020 (2001).

32. A. A. Zavitsas, Properties of water solutions of electrolytes and nonelectrolytes. *J. Phys. Chem. B* **105**, 7805–7817 (2001).

33. Y. Xu, C. Wang, K. C. Tam, L. Li, Salt-assisted and salt-suppressed sol-gel transitions of methylcellulose in water. *Langmuir* **20**, 646–652 (2004).

34. R. H. Gerke, Temperature coefficient of electromotive force of galvanic cells and the entropy of reactions. *J. Am. Chem. Soc.* **44**, 1684–1704 (1922).

35. N. E. A. Shuhaimi, L. P. Teo, H. J. Woo, S. R. Majid, A. K. Arof, Electrical double-layer capacitors with plasticized polymer electrolyte based on methyl cellulose. *Polym. Bull.* **69**, 807–826 (2012).

36. N. E. A. Shuhaimi, S. R. Majid, A. K. Arof, On complexation between methyl cellulose and ammonium nitrate. *Mater. Res. Innov.* **13**, 239–242 (2009).

37. L. Zhang, T. Kim, N. Li, T. J. Kang, J. Chen, J. M. Pringle, M. Zhang, A. H. Kazim, S. Fang, C. Haines, D. Al-Masri, B. A. Cola, J. M. Razal, J. Di, S. Beirne, D. R. MacFarlane, A. Gonzalez-Martin, S. Mathew, Y. H. Kim, G. Wallace, R. H. Baughman, High power density electrochemical thermocells for inexpensively harvesting low-grade thermal energy. *Adv. Mater.* **29**, 1605652 (2017).

38. H. Im, T. Kim, H. Song, J. Choi, J. S. Park, R. Ovalle-Robles, H. Yang, K. D. Kihm, R. H. Baughman, H. H. Lee, High-efficiency electrochemical thermal energy harvester using carbon nanotube aerogel sheet electrodes. *Nat. Commun.* **7**, 10600 (2016).

39. R. Hu, B. A. Cola, N. Haram, J. N. Barisci, S. Lee, S. Stoughton, G. Wallace, C. Too, M. Thomas, A. Gestos, M. E. Cruz, J. P. Ferraris, A. A. Zakhidov, R. H. Baughman, Harvesting waste thermal energy using a carbon-nanotube-based thermo-electrochemical cell. *Nano Lett.* **10**, 838–846 (2010).

40. A. J. deBethume, T. S. Licht, N. Swendeman, The temperature coefficients of electrode potentials: The isothermal and thermal coefficients—The standard ionic entropy of the electrochemical transport of the hydrogen ion. *J. Electrochem. Soc.* **106**, 616–625 (1959).

41. Z. Karpas, Z. Berant, O. Shahal, Effect of temperature on the mobility of ions. *J. Am. Chem. Soc.* **111**, 6015–6018 (1989).
